# Exploring novel alkane-degradation pathways in uncultured bacteria from the North Atlantic Ocean

**DOI:** 10.1128/msystems.00619-23

**Published:** 2023-09-13

**Authors:** Mirna Vázquez Rosas Landa, Valerie De Anda, Robin R. Rohwer, Angelina Angelova, Georgia Waldram, Tony Gutierrez, Brett J. Baker

**Affiliations:** 1 Department of Marine Science, Marine Science Institute, University of Texas at Austin, Port Aransas, Texas, USA; 2 Instituto de Ciencias del Mar y Limnologia Universidad Nacional Autónoma de Mexico, Unidad Académica de Ecologia y Biodiversidad Acuática, Mexico City, Mexico; 3 Department of Integrative Biology, The University of Texas at Austin, Austin, Texas, USA; 4 School of Engineering and Physical Sciences, Institute of Mechanical, Process and Energy Engineering (IMPEE), Heriot-Watt University, Edinburgh, United Kingdom; California State University, Northridge, California, USA

**Keywords:** oil spills, stable-isotope probing (SIP), metagenomics, assembled genomes, hydrocarbon degradation

## Abstract

Microbes play a significant role in the degradation of petroleum hydrocarbons in the oceans, yet little is known about the native bacteria that metabolize hydrocarbons before an oil spill. The Faroe-Shetland Channel (FSC) is a deepwater subarctic region of the North Atlantic with prominent oil production and a diverse microbial community associated with the degradation of petroleum. Here, we combine DNA-based stable-isotope probing (DNA-SIP) with metagenomics to elucidate the metabolic underpinnings of native alkane-degrading bacteria from the FSC. From two ^13^C *n*-hexadecane SIP experiments using seawater from 5 and 700 m depths in the FSC, we obtained 42 metagenome-assembled genomes (MAGs) belonging to 19 genera, including two previously overlooked hydrocarbon-degrading bacteria, *Lentibacter* (Alphaproteobacteria) and *Dokdonia* (Bacteroidetes). Diversity surveys indicated *Lentibacter* were dominant members of the FSC, constituting up to 17% of these communities. Many of the SIP-enriched MAGs (20/42) encoded a complete alkane oxidation pathway, including alkane monooxygenase (AlkB), rubredoxin reductase (AlkT), and rubredoxin-2 (AlkG). Fourteen Aphaproteobacteria MAGs lacked AlkG for electron transfer. Instead, they encoded novel disulfide isomerases with iron-binding cysteine motifs conserved across rubredoxins. *Dokdonia* lacked AlkT and AlkG, however, their central alkane-degradation catabolic pathways were complete. We describe previously unrecognized bacteria capable of hydrocarbon degradation, including the dominant genera *Lentibacter*, which may continuously purge hydrocarbons released from oil exploration activities in the FSC. This advances the understanding of the diversity and physiologies of alkane degradation in the North Atlantic and provides evidence of new mechanisms used to metabolize alkanes.

**IMPORTANCE:**

Petroleum pollution in the ocean has increased because of rapid population growth and modernization, requiring urgent remediation. Our understanding of the metabolic response of native microbial communities to oil spills is not well understood. Here, we explored the baseline hydrocarbon-degrading communities of a subarctic Atlantic region to uncover the metabolic potential of the bacteria that inhabit the surface and subsurface water. We conducted enrichments with a ^13^C-labeled hydrocarbon to capture the fraction of the community actively using the hydrocarbon. We then combined this approach with metagenomics to identify the metabolic potential of this hydrocarbon-degrading community. This revealed previously undescribed uncultured bacteria with unique metabolic mechanisms involved in aerobic hydrocarbon degradation, indicating that temperature may be pivotal in structuring hydrocarbon-degrading baseline communities. Our findings highlight gaps in our understanding of the metabolic complexity of hydrocarbon degradation by native marine microbial communities.

## INTRODUCTION

Oil spills at sea are one of the most harmful anthropogenic pollution events, and the Deepwater Horizon spill is a testament to how profoundly the health of marine ecosystems and the livelihood of its coastal inhabitants can be impacted by spilled oil. Oil can spread for many miles in seawater, and its impact on marine ecosystems far exceeds spills on land. For example, the Deepwater Horizon oil spill (20 April 2010) released ca. 3.19 million barrels of crude oil into the Gulf of Mexico, contaminating an area of 62,159 km^2^ (1). While oil-spill response measures may help recover and clean up some of the spilled oil, the ultimate protagonists contributing to this process are hydrocarbon-degrading bacteria. The fate of oil in the environment is largely dictated by the presence and activities of these organisms, which are ubiquitous in the world’s oceans, often in low abundances (<1% of the total bacterial community), until they become strongly enriched in the event of an oil spill and play an essential role in restoring oil-affected ecosystems to their natural state (2, 3).

Much of what we know about the genes, enzymes, and pathways in aerobic hydrocarbon degradation by bacteria and other microbes is the result of a plethora of studies using cultured strains. With alkanes, which are the most abundant constituents of crude oil and many of its refined products (diesel, gasoline, etc.), their aerobic biodegradation begins with their oxidation to form alcohol by a complex of three enzymes: alkane monooxygenase (AlkB), rubredoxin reductase (AlkT), and rubredoxin-2 (AlkG). In subsequent steps, an alcohol dehydrogenase (AlkJ), aldehyde dehydrogenase (AlkH), and medium-chain-fatty-acid-CoA (AlkK) transform the oxidized alkanes into fatty acids, which are then channeled into beta-oxidation (4 – 11). The canonical pathways involved in alkane degradation encompass several key enzymes, including AlkBFGHJKL. AlkB is a non-heme diiron integral membrane protein that serves as a marker for bacteria capable of utilizing alkanes as their sole source of carbon and energy (12). The genomic location and organization of *alkBFGHJKL* genes exhibit significant variability among hydrocarbon-degrading bacteria. For instance, these genes have been identified in plasmids, as observed in *Pseudomonas putida GPo1*, or on the main chromosome, as observed in *Amycolicicoccus subflavus* DQS3-9A1 (13 – 15). In *Rhodococcus erythropolis SK121*, multiple copies of *alkB* have been discovered. In Actinobacteria such as *Streptomyces*, *Aeromicrobium*, *Gordonia*, and *Dietzia*, *alkB* has been identified fused with rubredoxin domains. This fusion has been proposed to potentially enhance the enzyme’s capacity for degrading longer-chain n-alkanes, expanding its ability to efficiently utilize a broader range of alkane substrates (14). The diverse arrangements of these genes suggest the existence of alternative genetic mechanisms for hydrocarbon degradation that have yet to be fully elucidated (16).

Studies exploring the hydrocarbon-degrading capabilities of bacterial communities are typically conducted during or after an oil spill incident and typically involve single-gene diversity surveys (15, 17). These studies lack genome-level characterization of the bacteria actively involved in utilizing the hydrocarbons. DNA-based stable-isotope probing (DNA-SIP) is a technique used to obtain the DNA of microbial populations that actively metabolize a target substrate which is isotopically labeled (often ^13^C) after the label has been sufficiently incorporated into their DNA (18). For instance, ^13^C-*n*-hexadecane and other isotopically labeled compounds can be used to enrich hydrocarbon-degrading organisms in natural communities (19). The ^13^C-enriched DNA recovered from a SIP experiment can then be used in metagenomics studies, such as to reconstruct the genomes (SIP-metagenomics) of members of the community that utilized the isotopically labeled substrate. To the best of our knowledge, this method has been used to characterize the physiologies of bacteria that responded to the Deepwater Horizon spill (20) and a chronically polluted marine environment (21). Hitherto, this SIP-metagenomics approach has not been applied to investigate baseline hydrocarbon-degrading communities in marine environments where there is no obvious source of oil spillage.

Here, we investigated the hydrocarbon-degrading bacterial communities of the Faroe-Shetland Channel (FSC), which is a deepwater region of the northeast Atlantic Ocean with a history of oil exploration and production spanning more than two decades, but which shows no apparent oil seepage or spillage. Recently, the first microbiological baseline was established for this deepwater subarctic region, which revealed the presence of putative and recognized hydrocarbon-degrading bacteria in surface and subsurface waters, several of which are uncultured taxa and for which there is no information on their hydrocarbon-degrading capabilities and other metabolic functions (22). Here, we used SIP-metagenomics to understand the metabolic mechanisms of some of these organisms in the baseline communities of the FSC. We obtained 42 metagenome-assembled genomes (MAGs) from these enrichments, including two bacterial lineages for which the hydrocarbon-degradation pathway had not been described previously.

## MATERIALS AND METHODS

### Field sampling

Water samples were collected from location FIM6a (60° 38′N, 4° 54′W) at depths 5 and 700 m during a research cruise on the MRV *Scotia* between 24 April to 9 May 2014. This sampling site lies on the Fair Isle-Munken line (23) near the Foinaven oil field development area, approximately 3 and 9.3 nautical miles away from the Petrojarl Foinaven and Glen Lion production facilities, respectively. Seawater samples (3 L volumes) were collected using 10 L Niskin water flasks mounted on a CTD (conductivity, temperature, and depth) carousel in accordance with *MRV Scotia’s* sampling procedures (23). CTD casts confirmed these samples were taken from two distinct water masses, the Modified North Atlantic Water and the Norwegian Sea Arctic Intermediate Water (24). Immediately after recovery, a portion of the collected seawater was used to rinse, at least three times, two Nalgene carboys (10 L each; acid-washed, acetone-rinsed, and dried) prior to filling and immediately storing at 10°C onboard the vessel until return to the laboratory at Heriot-Watt University for immediate use for SIP experiments.

### SIP incubations

Prior to the preparation of the SIP incubations, each of the two seawater samples (collected at 5 and 700 m depths) were processed to remove dissolved organic carbon/matter that could potentially act as an alternative carbon source and redirect microbial activity away from the labeled substrates during SIP. For this, 800 mL of each sample was filtered through 0.22 µm MCE filters (47 mm diameter; Millipore Sigma). The bacterial biomass was collected on the filters and rinsed with a few milliliters of sterile synthetic seawater medium ONR7a (25) and then re-suspended in sterile 40 mL of the ONR7a to act as the inoculum for SIP.

To prevent hydrocarbon sorption, SIP incubations were conducted using 125 mL sterilized glass screw-top Erlenmeyer flasks with foil-lined lids. ONR7a medium was used in these incubations because, as previously indicated, we wanted to prevent the introduction of exogenous and potentially bioavailable carbon sources. Each flask contained 16 mL of ONR7a medium, 1 mg of labeled (^13^C) and/or unlabeled *n*-hexadecane, and 4 mL of inoculum for incubations utilizing either the surface 5 m depth water or the deep water (700 m depth) inoculum. SIP experiments were set up with two hydrocarbon substrates (U-^13^C) and unlabeled (^12^C) *n*-hexadecane of >99% purity according to Sigma-Aldrich. Duplicate flasks were prepared with 1 mg of U-^13^C-labeled *n*-hexadecane, and a second set of duplicates was made with 1 mg of the respective unlabeled hydrocarbon. An additional set of triplicate flasks was prepared containing unlabeled *n*-hexadecane to monitor its disappearance by gas chromatography-mass spectrometry (GC-MS). Samples were periodically taken from these flasks for DNA extraction and subsequent measurement by quantitative PCR (described below) to determine the abundance of target organisms identified through SIP. An additional set of triplicate flasks was prepared for each SIP experiment to act as acid-killed controls (pH < 1) containing unlabeled hydrocarbons and amended with 750 µL of 85% phosphoric acid. All flasks were incubated on an orbital shaker (150 rpm) in the dark at 21°C. Tracking the disappearance of the hydrocarbon in the triplicate containers using GC-MS was used to determine the end point of each SIP experiment. While DNA was extracted using a standard protocol (26) from the total volume of the paired flasks amended with the (U-^13^C) hydrocarbon and the corresponding paired set containing unlabeled hydrocarbons (Fig. S1).

### DNA gradient ultracentrifugation and identification of labeled 16S rRNA genes

Isopycnic ultracentrifugation of DNA from each of the SIP experiments using the 5 or 700 m seawater samples amended with ^13^C-labeled *n*-hexadecane resulted in the visual separation of two bands (~1 cm apart from each other) that were in the lower half of the polyallomer tubes. These bands were consistent with the expected location of the “heavy” and “light” SIP-DNA bands. With the respective acid-killed controls, it later became apparent that instead of concentrated acid, a very dilute concentration had been added to these control incubations and which was apparent from the degradation data (Fig. S1). This was insufficient to completely suppress microbial activity, which explains why some hydrocarbon degradation was observed in these controls. However, this did not affect our assessment in monitoring the degradation of the *n*-hexadecane in the “live” incubations, nor did it prevent us from determining when to terminate these experiments for DNA extractions from the ^13^C incubations. At the end of 5 days, duplicate ^13^C incubations were subjected to DNA extractions followed by isopycnic ultracentrifugation to isolate the ^13^C-enriched “heavy” DNA for subsequent molecular and bioinformatic analysis. Subsequent denaturing gradient gel electrophoresis (DGGE) analysis of the fractions derived from the labeled incubations performed for each of the two water samples showed clear evidence of isotopic enrichment of DNA. This is evident from the distinct separation of the ^13^C-enriched and unenriched DNA fractions by DGGE (Fig. S2) and by the distribution of qPCR-quantified 16S rRNA gene sequences (Fig. S3). The combined fractions containing ^13^C-enriched DNA from each of these ^13^C incubations were used to construct 16S rRNA gene clone libraries. The same analysis was performed on duplicate ultracentrifuge tubes from each of the ^13^C incubations to confirm our results (data not shown).

### Cesium chloride gradient ultracentrifugation and identification of ^13^C-enriched DNA

Total extracted DNA from each of the duplicate unlabeled and ^13^C-labeled incubations was added to cesium chloride (CsCl) solutions (1.68 g/mL), and the ^13^C-enriched and unenriched DNA was separated by isopycnic ultracentrifugation and gradient fractionation as described in reference 27 with the following modifications. Before heat-sealing the polyallomer tubes, approximately 3 mL of mineral oil was applied to the top of each CsCl solution. The tubes were then ultra-centrifuged for 40 h using a fixed-angle rotor 70.1Ti (Beckman Coulter) at 187,000 *× g* at 20°C with a BC Optima L-100 XP ultracentrifuge (Beckman Coulter).

DGGE was conducted on each fraction from the SIP tubes after isopycnic ultracentrifugation to visualize and confirm the separation of DNA. For this, amplification of each fraction was carried out using PCR as described by reference 28 with bacterial primers 341f (5′-CCTACGGGAGGCAGCAG-3′) and 534r (5′-ATTACCGCGGCTGCTGG-3′), the forward of which contained a 40 nucleotide GC clamp (5′-CGCCCGCCGCGCGCGGCGGGCGGGGCGGGGGCACGGGGGG-3′) (29). PCR products were verified on a 1.5% (wt/vol) agarose gel alongside a HindIII DNA ladder (Invitrogen, Carlsbad, CA, USA). DGGE was performed using 6.5% acrylamide gels containing a denaturant range of 30–70% (100% denaturant contains 7.0 M urea and 40% molecular-grade formamide). After electrophoresis for 16 h at 60°C and 60 V, gels were stained with ethidium bromide (1:25,000 dilution) for 15 min and then imaged with an InGenius3 gel imaging system (Syngene) and accompanying software to crop the gel images to only the regions displaying bands. The ^13^C-enriched heavy DNA fractions were selected based on the DGGE evidence, which is discussed below.

### 16S rRNA gene libraries of ^13^C-enriched DNA

Using general bacterial primers 27f and 1,492r, two 16S rRNA clone libraries (each comprising 96 clones) were generated from combined fractions containing the ^13^C-enriched DNA from each SIP experiment (29). PCR products were cloned using the TOPO-TA cloning kit for sequencing (Thermo Fisher Scientific). Clones were partially sequenced by GeneWiz (UK) using primer 27f. After excluding vector sequences, poor-quality reads, and chimeras, the clone sequences were classified into operational taxonomic units (OTUs) using a sequence identity threshold of 97%. A representative clone sequence was selected from each dominant OTU identified in each of the libraries and used to obtain a near-complete 16S rRNA gene sequence (>1,400 bp). Sequences were edited and assembled using Consed/Phred/Phrap (30). BLASTn searches and RDP-II were used to check for close relatives and phylogenetic affiliation (Fig. S4).

### Real-time quantitative PCR

To quantify sequences of the dominant OTUs, primers for real-time quantitative PCR (qPCR) were developed using AliView (31) and the NCBI Primer Blast online tool (32). Primer specificity was confirmed with the NCBI Primer-BLAST tool. The optimal annealing temperature of each primer pair was determined using an Applied Biosystems (Foster City, CA, USA) Mastercycler gradient thermal cycler. A plasmid containing a representative sequence that had been linearized with an appropriate restriction endonuclease and purified with the QIAquick nucleotide removal kit (Qiagen, Valencia, CA, USA) was used as a template for these reactions, and for the construction of respective standard curves for quantitative PCR. The final set of primers used can be found in Table S1.

Purified DNA from time-series incubations with unlabeled hydrocarbon was quantified using a NanoDrop ND-3300 fluorospectrometer (Thermo Scientific) and the Quant-iT Picogreen double-stranded DNA (dsDNA) kit (Invitrogen). From each SIP experiment, only one replicate of the duplicate ^12^C- and ^13^C-labeled incubations was selected for downstream analyses based on fractions containing the highest amount of total DNA. Using the qPCR primers designed to quantify sequences of the dominant OTUs in each separated SIP fraction, single reactions were performed on each triplicate DNA extraction (from triplicate samples) from the time series containing unlabelled hydrocarbons.

### Phylogenetic tree of the ^13^C-enriched community

The 16S rRNA genes of the SIP-identified sequences were assembled by using the program Sequencher 5.3 (GeneCodes Corp., Ann Arbor, MI, USA). The consensus sequences were submitted to GenBank and checked for close relatives and phylogenetic affiliation using BLASTn. The search results served as a guide for tree construction, and additional related 16S rRNA sequences found via BLASTn search were obtained from GenBank. The software package MEGAX (version 10.2.4) was used to align the sequences using MUSCLE and to construct a neighbor-joining tree with Jukes–Cantor correction. The tree was bootstrapped 1,000 times, and gaps in the alignment were ignored. *Roseibacillus ishigakijimensis* strain MN1-741 (NR041621), *Verrucomicrobium spinosum* strain DSM 4136 (NR026266), and *Haloferula chungangensis* strain CAU 1074 (NR109435) were used as an outgroup.

### Metagenomic sequencing and assembly of ^13^C-enriched DNA from SIP

Illumina library preparation, sequencing, and assembly of four samples were completed by the Joint Genome Institute (JGI) (33, 34). Data are available under project IDs 3300039448, 3300039456, 3300039449, and 3300040958. The four samples represent the heavy (^13^C-enriched) DNA from each of the duplicate SIP incubations using the 5 and 700 m seawater inoculum. Paired-end sequencing was performed on an Illumina NovaSeq 6000 platform with an average insert size of 241 and fragments of 300 bps. Raw reads were quality filtered following BBtools v35.74 (35) and assembled with metaSPAdes v3.14.1 (36) (Tables S2 and 3). Coverage information was obtained by mapping all high-quality reads of each sample against the assembly using the BWA-MEM v0.7.12 algorithm in paired-end mode (37).

### Genome binning

Assembled metagenomic data (contigs > 2,000 bp) was binned using MetaBAT2 v2.12.1 (38), and CONCOCT v1.1.0 (39), and resulting MAGs were combined using DAS Tool v1.1.2 (40). First, each of the mapping files was summarized using jgi_summarize_bam_contig_depths and then MetaBAT2 was run using the following settings: --minCVSum 0 --saveCls -d -v --minCV 0.1 m 2000 and CONCOCT as follows: --clusters 400 --kmer_length 4 --length_threshold 3000 --seed 4 --iterations 500. A scaffold-to-bin list was prepared for each of the two binning tools, and the DAS Tool ran on each of the eight scaffold files as follows: DAS_Tool -i Concoct.scaffolds.tsv, Metabat.scaffolds.tsv -l concoct,metabat -c assembly.contigs.fasta –debug -t –write_bins 1 –search_engine blast. The accuracy of all the MAGs was evaluated by calculating the percentage of completeness and gene duplication using CheckM v1.0.5 (41) (Table S4). MAGs greater than or equal to 50% of completeness and <10% gene duplications (according to checkM) were used in this study. MAG relative abundance was calculated as previously described in (42) using the bin_abundance.py script from MetaGaia (https://github.com/valdeanda/MetaGaia) (Table S5).

### Phylogenetic reconstruction, taxonomy, and the pangenome of the *Oceanobacter-*related bacteria

GTDB-Tk v1.5.0 (43) was used for preliminary taxonomic identification of the 42 individual genomes (Table S6). This information was used to explore community structure among metagenomic replicates by performing an NMDS analysis using the vegan package (44) implemented in R (Fig. S5). Then 37 conserved marker proteins (mainly ribosomal) were extracted using PhyloSift v1.0.1 (45). We used the 30S ribosomal protein S2 protein of the 37 marker proteins identified to perform a BLASTp search against the RefSeq database (date of search 14 September 2021) to obtain the closest publicly available genomes. Based on GTDB-tk results of predicted taxonomy, we also downloaded 30 genomes from Actinobacteria from RefSeq as an outgroup. We also extracted the 37 marker genes using phylosift for these reference genomes and added them to our analyses. An alignment of the extracted assembled MAGs and reference genomes was generated using MAFFT v7.487 (46) as follows: –globalpair –maxiterate 16 –reorder. The alignment was trimmed using trimAL (47) -automated1. The phylogeny was constructed with RAxML v8.2. (48) as follows: raxmlHPC-PTHREADS-AVX -f a -m PROTGAMMAAUTO -N autoMRE -p 12,345 × 12,345. Finally, we performed a pangenome analysis, including several reference genomes from *Oceanobacter*-related bacteria (49), using the software get_homologues (50) and the OrthoMCL algorithm to find the orthologue clusters.

### Metabolism reconstruction

Gene prediction for individual MAGs was performed using Prodigal v2.6.3 (51). Predicted genes of individual MAGs were further characterized using KofamScan (52 ), and InterProScan v5.31-70.0 (53) using default parameters. For KofamKOALA, only hits above the predefined threshold for individual KOs were selected. We then implemented an open-source R package called rbims (https://github.com/mirnavazquez/RbiMs) that reads, evaluates, and visualizes the annotation profile output derived from KofamScan. We used the function “read_ko” to calculate the abundance of each KO within each MAG and the function “mapping_ko” to link each KO to other KEGG database features. We also linked each KO to the rbims database, which includes a definition of the aerobic hexadecane degradation pathway (20) (Table S7). We performed functional annotation based on KEGG for enzymes that are exclusively found in our MAGs belonging to the Oceanobacter-related bacteria but absent in the reference genomes of *Oceanobacter*. These orthologues were then annotated using the KEGG database, following the same methodology as described previously (see Table S8). Our search for hydrocarbon-related genes encompassed two distinct approaches. Firstly, we utilized the CANT-HYD database (Calgary approach to ANnoTating HYDrocarbon degradation genes) (54) by employing the --cut_nc noise cut-off (Table S9) to identify experimentally validated monooxygenases involved in aerobic hydrocarbon degradation. Secondly, to expand our search, we conducted BLASTp searches targeting homologs of transporters known to participate in hydrocarbon degradation, as documented in *Alcanivorax dieselolei* (55) (Table S10). By employing both methods, we ensured a comprehensive exploration of hydrocarbon-related genes using a combination of experimentally validated and homology-based approaches.

### Phylogenetic reconstruction of Alk proteins

The metagenome entropy-based score MEBS v1.2 (56) was used to search the protein families associated with aerobic alkane degradation: AlkB (PF00487), AlkT (PF07992, PF18113), AlkG (PF00301), AlkJ (PF00732, PF05199), and AlkH (PF00171). We performed a BLASTp search against the non-redundant database from NCBI (14 September 2021) and queried all the proteins previously identified by MEBS, and used the first hits as references for phylogenetic reconstructions. We also downloaded from the UniProt database (The UniProt Consortium) the sequences from well-characterized AlkB proteins: Q0VTH3, Q0VKZ3, O31250, and P12691; AlkG: Q9HTK7, Q9HTK8, P00272, Q9WWW4, and Q0VKZ2; AlkT: P17052, P42454, Q0VTB0, Q9HTK9, and Q9L4M8; AlkJ: Q00593, and Q9WWW2; AlkH: P12693. Identified alkane degradation coding genes and publicly available references were concatenated and aligned using MAFFT v7.487 (46) as follows: –globalpair –maxiterate 16 –reorder. Phylogeny was generated using IQtree v1.6.12 (57) with the following parameters: -alrt 1000 -bb 1000 -bnni (Fig. S6 to S9).

### Operon analysis and AlkG-like alignment

We used the operon mapper web server (58) to identify the operons where AlkB was present. We extracted the sequences that belonged to the thiol-disulfide isomerase family (COG1651) found next to the AlkB in 17 MAGs. We also downloaded from UniProt (The UniProt Consortium) and NCBI well-characterized AlkG proteins: P00271, Q9WWW4, Q0VKZ2, WP_138436252.1, WP_161463810.1, WP_089423380.1, WP_084394766.1, WP_015486580.1, Q9HTK8, and Q9HTK7. The reference and COG1651 sequences were aligned using MAFFT v7.487 (46) (–globalpair –maxiterate 16 –reorder).

## RESULTS

### Microbial diversity and abundance of clone libraries

To determine the taxonomic diversity of the enriched community from SIP, we sequenced clone libraries to obtain full-length 16S rRNA gene sequences (>1,400 bp) see Table 1. The results showed that a small number of OTUs comprised most of the SIP-enriched communities, with four OTUs accounting for 77.6% of the 5 m community and six OTUs comprising 61.7% of the 700 m community (see Table 1). All other OTUs at each depth represented less than 5% of total sequences and were not further analyzed. The abundant OTUs were distributed among three phyla: Gammaproteobacteria, Alphaproteobacteria, and Bacteroidetes. Specifically, the Gammaproteobacteria were comprised of five genera *Alcanivorax*, *Marinobacter*, *Glaciecola*, *Thalassolituus*, and *Oleibacter*, which has recently been proposed to be reclassified as *Thalassolituus* (49). The Alphaproteobacteria comprised two genera: *Lentibacter* and *Phaeobacter*, and the Bacteroidetes comprised the genus *Dokdonia* (see Table 1). At 5 m, members of *Alcanivorax*, *Lentibacter*, *Thalassolituus*, and *Oleibacter* were found, while at 700 m, we found *Oleibacter*, *Alcanivorax*, *Marinobacter*, *Phaeobacter*, *Glaciecola*, and *Dokdonia*.

**TABLE 1:** Microbial diversity and abundance of 16S rRNA gene sequences based on PCR amplification and clone libraries from the SIP enrichments^*a*^

OTU no.	Rep. seq.^*b*^	Closest BLASTn match^*c*^	Accession no.	Library (%)^*d*^
SIP using surface (5 m depth) seawater:				
OTU-2.14	HEX-5m-2.14	*Alcanivorax borkumensis* (99.32%)	NR074890	31.9
OTU-2.6	HEX-5m-2.6	*Thalassolituus oleivorans* (99.67%)	NR102806	22.3
OTU-1.1	HEX-5m-1.1	*Lentibacter algarum* (99.56%)	NR108333	14.9
OTU-2.4	HEX-5m-2.4	*Oleibacter marinus* (96.23%)	NR114287	8.5
SIP using subsurface (700 m depth) seawater:				
OTU-3.27	HEX-700m-3.27	*Oleibacter marinus* (96.36%)	NR114287	17
OTU-4.22	HEX-700m-4.22	*Alcanivorax borkumensis* (98.73%)	NR074890	12.8
OTU-3.15	HEX-700m-3.15	*Marinobacter algicola* (98.77%)	NR042807	9.6
OTU-4.3	HEX-700m-4.3	*Phaeobacter arcticus* (98.36%)	NR043888	8.5
OTU-3.32	HEX-700m-3.32	*Glaciecola nitratireducens* (95.33%)	NR074628	8.5
OTU-3.32	HEX-700m-3.32	*Glaciecola pallidula* (95.33%)	NR117119	8.5
OTU-4.12	HEX-700m-4.12	*Dokdonia genika* (95.42%)	NR041272	5.3

^*a*^ SIP with (U-^13^C) *n*-hexadecane.

^*b*^ Representative sequence for each OTU.

^*c*^ Results are to the closest type strain; percentage similarity shown in parentheses.

^*d*^ Total number of sequences in each of the ^13^C-enriched DNA clone library from the two *n*-hexadecane SIP incubations was 48. A 97% cutoff was used to classify sequences to an OTU.

**Fig 1:**
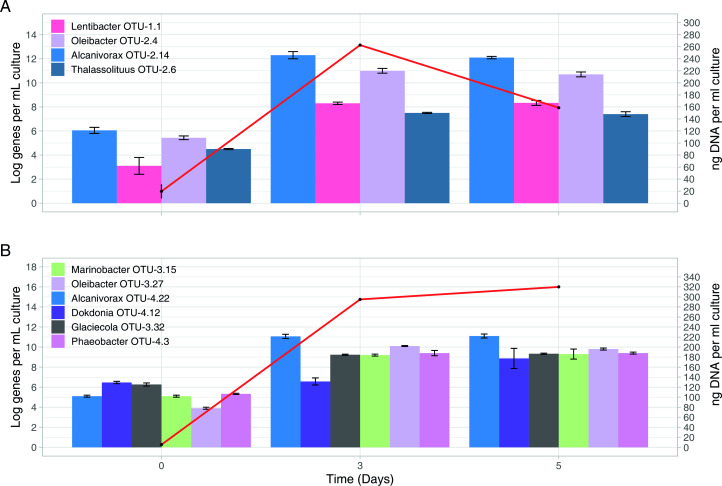
Microbial abundance based on qPCR of the 16S rRNA gene. Bars represent the average and standard deviation of results from triplicate qPCRs measuring the abundance of group-specific 16S rRNA genes. Points represent the mean and standard deviation of triplicate measurements of the total mass of DNA per sample. (**A**) The absolute abundance of *Lentibacter* (OTU-1.1), *Oleibacter* (OTU-2.4), *Alcanivorax* (OTU-2.14), and *Thalassolituus* (OTU-2.6) during incubation of the sea surface (5 m). (**B**) The absolute abundance of *Marinobacter* (OTU-3.15), *Oleibacter* (OTU-3.27), *Dokdonia* (OTU-4.12), *Glaciecola* (OTU-3.32), *Alcanivorax* (OTU-4.22) and *Phaeobacter* (OTU-4.3) during incubation of the 700 m water with unlabeled *n*-hexadecane.

**Fig 2:**
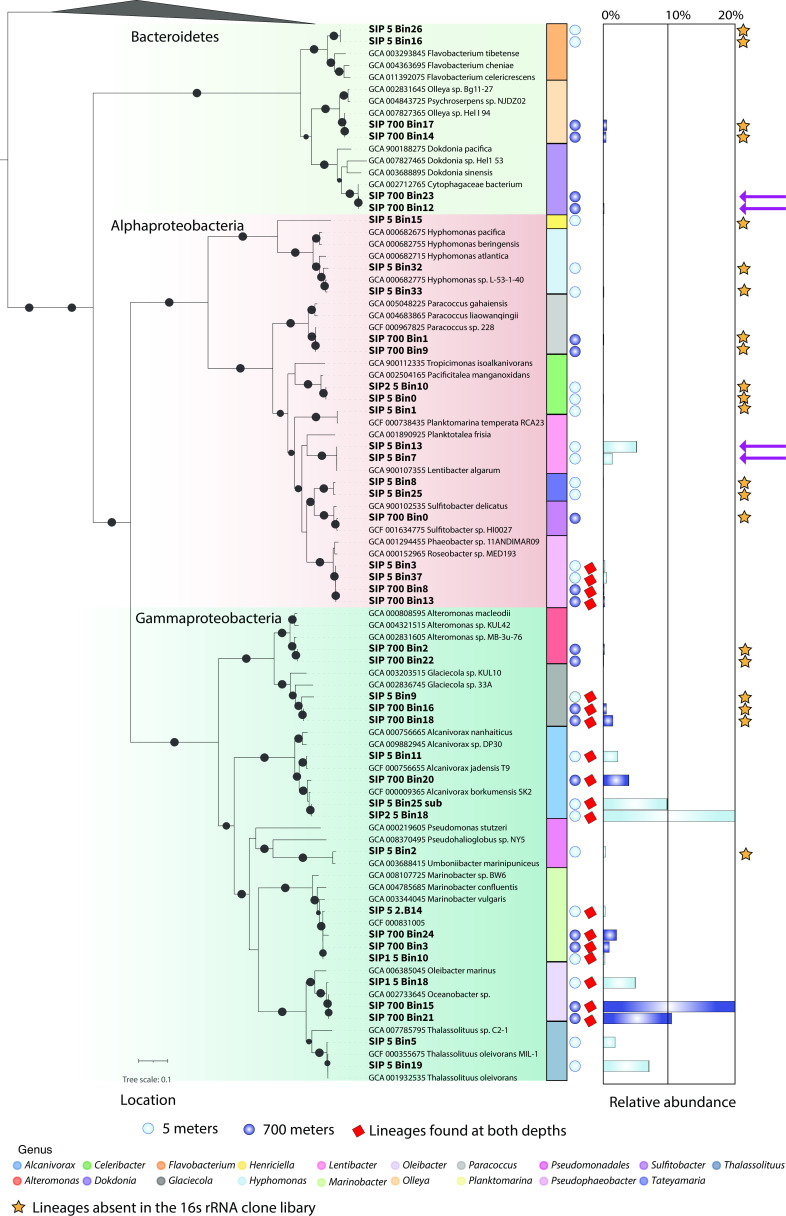
Phylogenetic reconstruction, distribution, and abundance of MAGs. A maximum-likelihood phylogenetic reconstruction of 37 conserved proteins of the 42 MAGs recovered from the SIP experiments. The black circles within the phylogeny indicate bootstrap values over 80%. The clade color indicates taxonomy. Green is Bacteroidetes, red is Alphaproteobacteria, and blue is Gammaproteobacteria. Color bars indicate taxonomy at the genus level. Dark blue and light blue circles represent the depth; dark blue from 700 m and light blue from 5 m. Red squares indicate MAGs found at both depths. The bars represent the abundance of each MAG within the metagenomic samples. The arrows point to the first assembled MAGs of those lineages recovered from a SIP enrichment experiment, and the stars represent the lineages identified through metagenomics but absent in the 16S rRNA clone libraries.

We used qPCR to determine the abundance of members of the enriched community (Fig. 1). During incubations of the 5 m water sample with unlabeled *n*-hexadecane, we observed a significant increase in the 16S rRNA gene copy numbers for several bacterial groups. Specifically, the 16S rRNA gene copy numbers for *Thalassolituus* OTU-2.6, *Lentibacter* OTU-1.1, *Alcanivorax* OTU-2.14, and *Oleibacter* OTU-2.4, increased by approximately 9, 16, 18, and 17 orders of magnitude, respectively, after 5 days (Fig. 1A). In the 700 m water sample, a marked increase in the 16S rRNA gene copy numbers were observed for six OTUs after 5 days of incubation with unlabeled *n*-hexadecane: *Dokdonia* OTU-4.12 (6 orders of magnitude), *Glaciecola* OTU-3.32 (10 orders), *Phaeobacter* OTU-4.3 (12 orders), *Marinobacter* OTU-3.15 (13 orders), *Alcanivorax* OTU-4.22 (18 orders), and *Oleibacter* OTU-3.27 (20 orders) (Fig. 1B). These results suggest that these bacterial groups have the metabolic capacity to utilize *n*-hexadecane and can thrive under these conditions. In the two SIP experiments, an increase in the 16S rRNA gene copy numbers of these OTUs provides further confirmation of their enrichment on the *n*-hexadecane as a growth substrate. These increases also coincided with an increment of the total concentration of DNA as a proxy for cell growth (Fig. 1). This, along with the disappearance (biodegradation) of the hydrocarbon and the appearance of the 16S rRNA genes of these organisms in the most heavily ^13^C-enriched DNA fractions, suggests that these organisms performed a primary role in the degradation of the *n*-hexadecane. Finally, qPCR showed that at 5 m depth, *Alcanivorax* OTU-2.14 was the most abundant genus during all 3 days of the experiment. At 700 m depth, *Dokdonia* OTU-4.12 was the most abundant during day 1, and *Alcanivorax* OTU-4.22 was the most abundant for the remaining 2 days (Fig. 2). This suggests that *Alcanivorax* is key to the alkane degradation process but that distinct *Alcanivorax* OTUs carry out alkane degradation at different depths.

### Microbial diversity and abundance of MAGs

To explore the genomic diversity and metabolic pathways of the hydrocarbon-degrading bacteria from the FSC, we assembled *de novo* four metagenomes (~400 Gb) from the 5 and 700 m SIP enrichments (two from each depth) and reconstructed 42 MAGs (Fig. 2). We obtained 24 and 18 MAGs from 5 and 700 m, respectively. The MAG sizes range from 1.76 to 4.88 Mb, with an average completeness of 94%, lowest completeness of 51%, and maximum gene redundancy of 10%. Phylogenomic analysis of 37 concatenated marker proteins (mainly ribosomal) revealed that these genomes belong to Bacteroidetes, Alphaproteobacteria, and Gammaproteobacteria (Fig. 2)*,* which is consistent with our 16S rRNA gene phylogenies (Fig. S4). Interestingly, 21 MAGs belonging to 11 genera were not recovered by the 16S rRNA gene clone libraries (stars in Fig. 2), suggesting that 16S rRNA gene-based approaches may overlook important hydrocarbon-degrading bacteria.

Of the 42 recovered MAGs, representatives of *Alcanivorax*, *Glaciecola*, *Marinobacter*, *Oleibacter*, and *Pseudophaeobacter* were obtained from both depths (blue dots in Fig. 2). *Pseudophaeobacter*, for example, was also identified via 16S RNA; however, it was classified as *Phaeobacter*. At 5 m, we recovered MAGs belonging to *Flavobacterium*, *Henricella*, *Hyphomonas*, *Celeribacter*, *Planktomarina*, *Lentibacter*, *Teteyamaria*, and *Pseudomonadales*, while at 700 m, we additionally recovered MAGs belonging to *Alteromonas*, *Olleya*, *Dokdonia*, *Paracoccus*, and *Sulfitobacter*. Notably, these are the first assembled genomes of *Dokdonia* and *Lentibacter* obtained from hydrocarbon SIP enrichment experiments.

Abundance estimations based on MAG genomic coverage indicated that the most abundant genera at 5 m were *Alcanivorax*, *Thalassolitus*, *Oleibacter*, and *Lentibacter* (bar plots in Fig. 2). This is consistent with the qPCR 16S rRNA analysis conducted in parallel (Fig. 1). Interestingly, the most dominant MAG in the SIP enrichment from 700 m *-Oleibacter-* has not been previously reported in FSC waters (22), suggesting that is likely present at low abundance in the baseline communities. *Lentibacter* has been previously described as a predominant genus in the baseline FSC water column (22, 59 – 61) that becomes enriched in the presence of crude oil (62, 63). However, alkane degradation and its pathways have not previously been experimentally confirmed in it. To identify the alkane degradation capabilities of these bacteria, we looked for genes that compose the canonical alkane degradation pathway.

### The alkane degradation pathway in FSC bacteria

**Fig 3:**
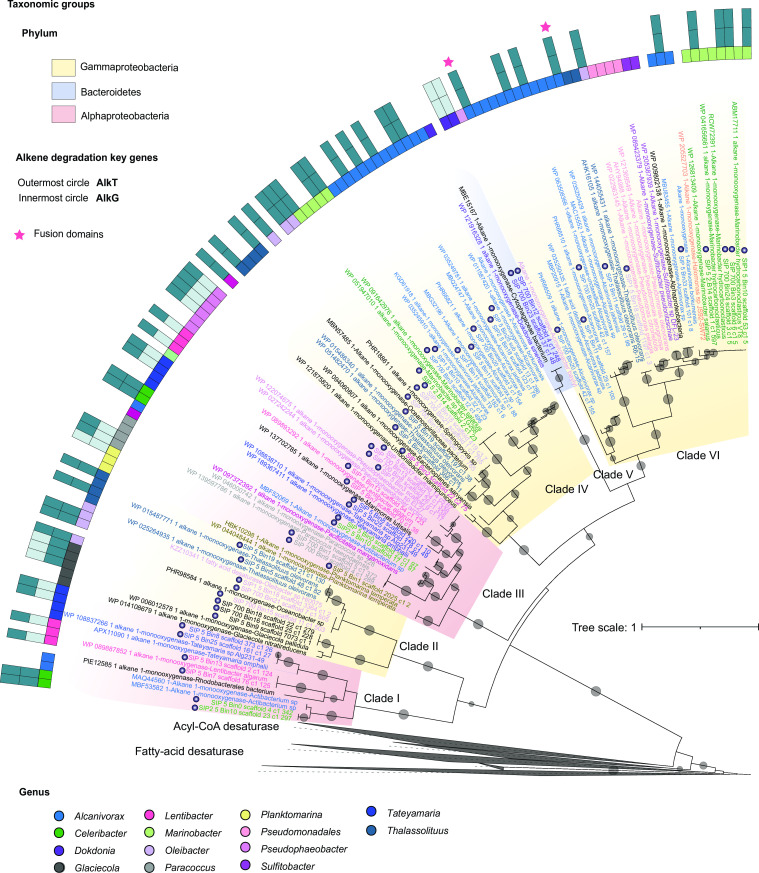
Diversity of AlkB and the distribution of AlkGT. Generated using RAxML (48) maximum-likelihood from the 42 FSC SIP-MAGs (see Materials and Methods). Gray circles within the branches indicate bootstrap values >80%, where the smallest circle equals 80% and the biggest 100%. The sequences of AlkB fall into SIX clusters (I–VI). Clades are color-coded according to the taxonomic group each sequence belongs. Yellow, Gammaproteobacteria; pink, Alphaproteobacteria; purple, Bacteroidetes. Label colors represent the genera to which each sequence belongs. Inner dots upon the labels show the sequence obtained in this study. The outer circles represent the presence or absence of rubredoxin (AlkT) and rubredoxin reductase (AlkG). The stars show the sequences with fusion domains. AlkB was present in 35 of the 42 MAGs.

To understand the metabolic pathways involved in hydrocarbon degradation, we searched for genes predicted to encode proteins involved in aerobic alkane utilization. We searched in the SIP-MAGs for homologs of AlkB and performed a phylogenetic reconstruction of these proteins. This revealed six distinct phylogenetic clusters that we named Clades I–VI (Fig. 3). All Gammaproteobacteria and Alphaproteobacteria had multiple copies of the AlkB gene. The Gammaproteobacteria *Alcanivorax* had two copies (Clades IV and VI), the Alphaproteobacteria *Lentibacter*, *Teteyamaria*, and *Celeribacter* had two copies (Clades I and III), and the Gammaproteobacteria *Thalassolituus* and *Oleibacter* had three copies (Clades II, IV, and VI). Multiple copies of AlkB gene suggest a high metabolic potential for alkane degradation. Furthermore, our pangenome comparison of the Oceanobacter-related bacteria revealed that these enzymes are absent in Oceanobacter reference genomes, confirming their presence as a trait of the *Thalassolituus* lineage (Fig. S11).

To incorporate the alkane molecule, AlkB requires rubredoxin (AlkG) and rubredoxin-reductase (AlkT) (64). Therefore, we also searched the MAGs for genes encoding these proteins (outer blue rings in Fig. 3). Sixteen Gammaproteobacteria MAGs had genes predicted to encode AlkGT (Clades II, IV, and VI), suggesting that they perform alkane degradation via the canonical pathway. In addition to lacking AlkBGT, seven MAGs belonging to the Gammaproteobacteria; *Alteromonas*, Bacteriodetes (*Flavobacterium* and *Olleya*), and Alphaproteobacteria (*Sulfitobacter*) lacked AlkJK. Among these bacteria, two Alphaproteobacteria and *Henriciella* MAGs also lacked genes coding for AlkBGT. Furthermore, the two Alphaproteobacteria, *Henriciella*, and the seven Gammaproteobacteria MAGs were some of the lowest in abundance in the SIP enrichments (Fig. 2), suggesting that they lack the potential for hydrocarbon degradation. In contrast, some AlkB-encoding MAGs were abundant in the SIP enrichment but lacked either AlkG or AlkT, suggesting complete hydrocarbon degradation is achieved via the non-redundant capabilities of other bacteria encoding these other genes. The absence of certain genes in these bacteria could be due to the incompleteness of these MAGs, or it is also possible that there is a new enzyme that is interacting with AlkB, and we were not able to identify it.

The oxidation of alkanes involves multiple varieties of monooxygenases. These include propane monooxygenase (PrmAC), butane monooxygenase (PBmoBAC, SBmoXYZ), flavin binding monooxygenase (AlmA) for alkanes ranging from C_20_ to C_32_, and alkane hydroxylases such as Cyp153, which oxidize alkanes from C_5_ to C_13_. The long-chain alkane hydroxylase (LadA/B) can also oxidize alkanes ranging from C_15_ to C_36_. These enzymes differ in their ability to incorporate alkanes of different sizes (54).

**Fig 4:**
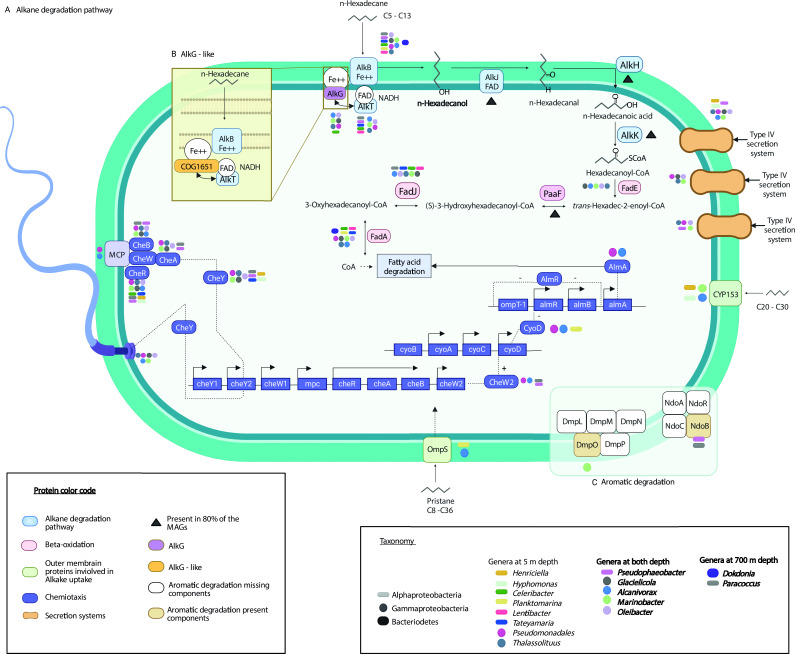
Overview of the alkane metabolism in the FSC. Overview of the alkane metabolism in the FSC community, highlighting the enzymes involved in hydrocarbon degradation. The diagram encompasses genes from different bacteria in the enrichments. The canonical alkane degradation pathway is in blue and emphasizes the beta-oxidation pathway in pink. The outer membrane proteins discovered in *Alcanivorax dieselolei* are shown in green, and the chemotaxis genes in purple (55). The main secretion systems are orange. Enzymes found in more than 80% of the MAGs are denoted by a triangle next to the enzyme. Below each key enzyme, the taxa containing that enzyme are displayed. The shapes used in the diagram represent the three phyla: Alphaproteobacteria (oval), Bacteroidetes (round squares), and Gammaproteobacteria (circle), while the different colors indicate distinct genera. The model in section B shows a potential new enzyme interacting with AlkB, while section C highlights enzymes involved in the degradation of aromatic compounds.

In the SIP-MAGs, we identified Cyp153 hydroxylase in *Marinobacter*, *Alcanivorax*, *Hyphomonas*, and *Henriciella* (Fig. 4). Additionally, we found a Flavin binding monooxygenase (AlmA) in *Alcanivorax* MAGs (Fig. 4). We also looked for other monooxygenases that typically participate in oxidizing aromatic compounds, and we identified one component of the toluene ortho monooxygenase enzyme (DmpO) in *Marinobacter*, as well as one component of the naphthalene-1,2 dioxygenase (non-NdoB) in *Pseudophaebocater* and *Paracoccus* (Fig. 4). The gene *ompS* in *A. dieselolei* has been described as an outer membrane protein involved in the degradation of pristine alkane (55). We found homologs to this protein in *Planktomarina* and *Alcalinivorax* MAGs. Finally, we identified genes related to flagella biosynthesis, secretion systems, and chemotaxis, primarily present in Gammaproteobacteria, indicating a potential community response towards *n*-hexadecane as previously observed for *Marinobacter* (65) and *Alcalinivorax* (55).

Since the genomes of these bacteria were isolated from waters unrelated to any documented occurrence of an oil spill, it is likely that they can utilize alternative energy sources in the absence of alkanes. Since the degradation of hydrocarbons may be facilitated by additional cellular processes, we searched for additional energy-generating processes in the MAGs. Interestingly, we identified denitrification genes (*napAB*; nitrate reductase, *norBC*; nitric oxide reductase, *nosZ*; nitrous-oxide reductase) in the *Dokdonia* genomes. Furthermore, Alphaproteobacteria MAGs contained genes that are predicted to encode the Sox enzyme complex involved in sulfur oxidation (SoxXYZABC) (66). In the absence of alkanes, key sulfur and nitrogen genes suggest that these bacteria are metabolically versatile and encode potential alternative mechanisms for energy generation (Fig. S12).

### Fusion domains and putative AlkG in Alphaproteobacteria

We identified two AlkB from *Alcanivorax* in Clade VI containing a fusion rubredoxin domain (stars in Fig. 3). This suggests that these genes encode a protein with a transmembrane domain whose cytoplasmic site already contains the AlkG rubredoxin domain (PF00301). Similarly, we found that *Celeribacter* AlkG sequences (SIP_5_Bin0_scaffold_9_c1_22 and SIP2_5_Bin10_scaffold_4_c1_43) have the AlkG and AlkT domains fused (PF00301 and PF07992), suggesting they can interact with AlkB without AlkT (Fig. S13).

**Fig 5:**
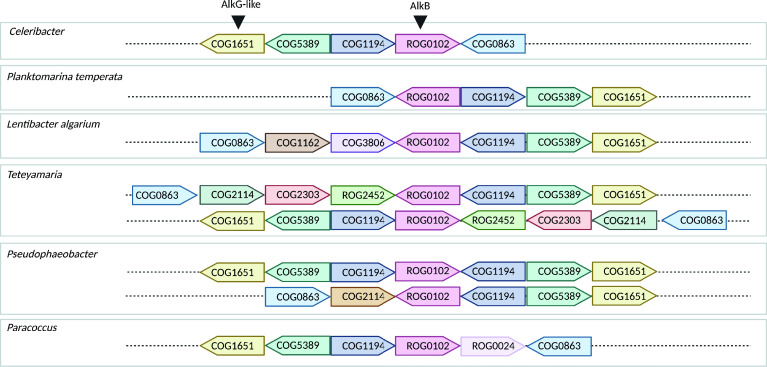
Characterization of genes neighboring *alkB* in Alphaproteobacteria belonging to Clade III. Predictions are based on the intergenic distance of surrounding genes and the functional relationships of their protein-coding products. The numbers inside the arrows represent COG IDs. Colored arrows in a pink show the AlkB gene (ROG0102), and in yellow, the AlkG-like gene (protein disulfide isomerase; COG1651). Arrows pointing to the right indicate that the gene is in the positive chain, whereas it is negative for arrows pointing to the left. The size of the arrows does not represent the size of the gene. Each row represents an organism. The rest of the genes correspond to: in light green, COG5389 (Protein of unknown function; DUF721); in dark blue, COG1194 (base-excision repair); in light blue, COG0863 (N-4 methylation of cytosine); in brown, COG1162 (GTPase activity); in purple, COG3806 (Anti-sigma factor), in dark green, COG2114 (Pfam Adenylate and Guanylate cyclase catalytic domain), in red, COG2303 (choline dehydrogenase activity), in green, ROG2452, in light brown, COG2114 (Pfam Adenylate and Guanylate cyclase catalytic domain), and light purple, ROG0024.

The FSC *Lentibacter* MAGs similarly had two copies of AlkB and lacked AlkG, yet, there was no evidence of fused domains like in *Alcanivorax* and *Celeribacter*. Therefore, we searched for additional potential electron carriers in these genomes. We examined the genes surrounding *alkB* in all the Clade III Alphaproteobacteria, which includes *Lentibacter*. We found that there are some genes with shared functional annotations in that genomic region (Fig. 5). We searched the proteins encoded by these genes for the conserved AlkG motif (Cys-X-X-Cys-Gly), which is the motif that interacts with AlkB (67). We identified this motif in a protein present in all 15 MAGs in Clade III annotated as a disulfide isomerase (COG1651) (see Fig. S11). The disulfide isomerase protein domain is involved in forming and regulating disulfide bonds in proteins such as DsbC (68). The presence of this motif, along with the operon analysis and the metabolic repertoire of enzymes in the alkane degradation pathway (AlkBTHJK) suggest the disulfide isomerase could be acting as a rubredoxin transferring electrons from AlkB to AlkT in Alphaproteobacteria (Fig. 4B).

## DISCUSSION

In this study, we used a multidisciplinary approach combining 16S rRNA analyses, ^13^C-*n*-hexadecane enrichments, and SIP-metagenomics to examine the microbial diversity and genetic mechanisms of alkane-degrading bacteria in the FSC, a subarctic deepwater region with an active oil industry presence. Our results revealed that the microbial communities varied by depth, with *Alcanivorax* dominating the surface waters (5 m) and *Oleibacter* being the most abundant alkane degrader in the subsurface (700 m). While our findings are based on laboratory-based enrichments, which may not always reflect how communities respond in their natural environment, our findings are consistent with previous studies that have reported the dominance of *Alcanivorax* in surface waters and the prevalence of *Oleibacter* in deeper waters. For example, a recent study analyzing the microbial communities in the FSC over a period of 2 years showed that *Alcanivorax* significantly increased in abundance in the upper water column (<175 m), and this was more prominent during the spring compared to the fall (from undetectable to 0.1% relative abundance) (22).

Two *Dokdonia* MAGs, at 700 m, were shown to be dominant at the beginning of the SIP enrichment. Previous studies have detected *Dokdonia* species in hydrocarbon-contaminated marine environments (69), but their hydrocarbon-degrading capabilities have not been substantiated. Here, we show for the first time that members of this genus are capable of hydrocarbon degradation. *Lentibacte*r is another lineage that has been shown to be predominant in the FSC water column (22, 59 – 61) that becomes enriched in the presence of crude oil (62, 63), but similarly to *Dokdonia*, their hydrocarbon-degradation capabilities for any member of this genus has not been previously reported. Here, we found *Lentibacter* MAGs account for 8% of the ^13^C-enriched communities from the 5 m SIP experiments, confirming their role in oil degradation. Earlier work assessing the bacterial diversity of the water column in the FSC over a 2-year period showed *Lentibacter* represented anywhere between 1% and 20% of the total community (22). Other genera, such as *Alteromonas* and *Sulfitobacter*, were also previously found in the FSC during the spring (22), but we were unable to find genomic-based evidence of any canonical alkane degradation pathway in these MAGs. This is not unexpected as not all members comprising each of these two genera possess the ability to utilize hydrocarbons.

We also found *Glaciecola* MAGs comprised 5% of the SIP enrichments at 5 and 700 m and coded all the enzymes required for alkane degradation (except AlkT). Isolates of *Glaciecola* from the North Sea—a waterbody adjacent to the FSC—have been shown to utilize hydrocarbons, including alkanes, as a sole source of carbon and energy (70), and representatives of this genus have been found enriched in laboratory cultures using FSC surface water amended with the soluble hydrocarbon fraction of crude oil (71). Furthermore, *Glaciecola* has been identified in pyrene-enrichments of sediment samples obtained at 500 and 1,000 m depths from the FSC (72). These findings suggest that *Glaciecola* could play a key role in hydrocarbon degradation in the event of a spill in the North Atlantic.

Previous *n*-hexadecane SIP experiments to identify the bacterial community that contributed to the degradation of the oil spilled into the Gulf of Mexico from the Deepwater Horizon spill (20), recovered MAGs affiliated with *Marinobacter*. While this genus is considered the most important of the generalist hydrocarbon degraders in the ocean, its members exhibit an almost exclusive preference for *n*-alkanes (73). *Marinobacter* has been found throughout the FSC water column (up to 0.2% relative abundance) ( 22, 74). Here, we recovered four *Marinobacter* MAGs evenly distributed across depths, though more abundant in the 700 m SIP experiment. Other less abundant lineages lacking AlkB, including *Flavobacterium*, *Henriciella*, *Alteromonas*, *Olleya*, and *Sulfitobacter*, have previously been found in oil-impacted ecosystems or implicated in oil degradation (75 – 78). *Flavobacterium*, among other methylotrophic organisms, has been found dominant in oil-impacted ecosystems, often succeeding after an initial enrichment by other bacteria, such as *Oceanospirillales* (17). *Sulfitobacter*, *Alteromonas*, and some *Flavobacterium* have been implicated in either or both alkane and aromatic hydrocarbon degradation (17, 79, 80), suggesting that these organisms may play a role in hydrocarbon degradation, either indirectly or in concert with other degraders (20).

Genomic-based metabolic inferences indicated that many of the FSC MAGs lacked *alkB* genes, which are often used as a biomarker to explore the diversity of bacteria capable of degrading hydrocarbons. Phylogeny revealed six taxonomic clades of AlkB distributed among Gammaproteobacteria, Alphaproteobacteria, and Bacteroidetes. Interestingly, most MAGs (except for *Dokdonia*) showed more than one version of AlkB, and the origin of these multiple copies could be gene duplication or acquisition via horizontal gene transfer (HGT) ( 81, 82). The AlkB gene is known to be horizontally transferred. For example, the OCT plasmid of *P. putida* which contains *alkB* was originally described in *Alcanivorax* and has been found distributed in genomes of different lineages (14, 83). Since the AlkB clades are composed of genomes from multiple genera, our analysis suggests that the most likely explanation for the multiple copies of the AlkB gene is HGT. Gene duplication has also been described before for *alkB*, mainly associated with multidomain versions and the capacity to use large alkanes of chain length from C14 to C30 (14, 84, 85).

The occurrence of AlkB enzyme across taxonomic lineages with unique genomic configurations and evolutionary histories suggests the possibility of alternative electron donors (i.e., AlkGT-like). Here, we identified a putative protein in Clade III Alphaproteobacteria MAGs that could interact with AlkB. This putative protein shares the AlkG-motif that interacts with AlkB. In *Celeribacter* MAGs, we also found a protein with the fusion domains of AlkGT, suggesting a new possibility of interaction with AlkB in Alphaproteobacteria.

Beyond the conventional alkane degradation pathway, our MAGs have a repertoire of metabolic capabilities that facilitate the breakdown and utilization of diverse alkanes. We identified other types of monooxygenases in Gammaproteobacteria and Alphaproteobacteria, such as the cyp153 hydroxylase in *Marinobacter*, *Alcanivorax*, *Hyphomonas*, and *Henriciella*, and the flavin-binding monooxygenase (AlmA) in *Alcanivorax* (as shown in Fig. 4). This suggests they may have been using different sizes of alkanes as an energy source. Additionally, we found genes involved in breaking down aromatic compounds, including genes coding for enzymes that help degrade naphthalene in Alphaproteobacteria and a component of the enzyme toluene monooxygenase in Gammaproteobacteria. Our results suggest that the ability to break down aromatic compounds may play a role in the niche partitioning among these bacteria.

The degradation of hydrocarbons by heterotrophic bacteria is challenging due to the very low solubilities of these compounds in water. Biofilms, including quorum-sensing mediated biofilms, have been proposed to facilitate access to hydrocarbon droplets, enabling bacteria to utilize these compounds as a carbon source (65, 86 – 88). In this study, we found genes related to access to hydrocarbon substrates. For example, all Gammaproteobacteria MAGs code genes related to motility, biofilm formation, and chemotaxis. Chemotaxis may provide a significant advantage, promoting a movement toward hydrocarbon molecules when the chemical concentration is low and allowing bacteria to avoid toxic hydrocarbons (89). For example, the type VI secretion system and genes related to chemotaxis have been observed in *Marinobacter* when exposed to *n*-hexadecane (90). Our results suggest that bacterial baseline communities in the FSC have genomic adaptations to survive in hydrocarbon-rich environments, as can occur during a major oil spill.

The functioning of marine ecosystems is closely linked to the cycling of key elements, such as nitrogen and sulfur. Our study provides evidence that hydrocarbon-degrading bacteria may play a role in these processes in the FSC. Specifically, we identified genes associated with denitrification in *Dokdonia* MAGs (from the incubations with 700 m depth seawater), suggesting that members of this genus may be involved in denitrification processes in the deep subsurface of the FSC. Since denitrification is a key process associated with oxygen-deficient zones, where some organisms can use alternative respiratory pathways when oxygen is depleted (91), hydrocarbon-degrading *Dokdonia* may have an advantage over other degraders during an oil spill. To the best of our knowledge, research specifically focused on the microbial response to crude oil in the FSC has consistently reported the presence of *Dokdonia*. However, none of the published studies have documented the enrichment of *Dokdonia* in these investigations. This is likely because those studies were conducted under well-aerated conditions, and of note, the FSC is a highly hydrodynamic area where oxygen is not known to be limited within the water column. However, in the event of a major spill in the deep waters of the FSC, hydrocarbon-degrading *Dokdonia* may be expected to play an important role when oxygen levels become limiting and when the activities of the aerobic oil-degrading population slow down or come to a halt, as occurred within the deep subsurface oil plume during the Deepwater Horizon oil spill (92). During that spill, the coupling of nitrogen fixation and growth on hydrocarbons (larger than methane and ethane) was, for the first time, shown to be closely intertwined (93 – 95). In addition to nitrogen, genes relevant to sulfur cycling have been reported enriched in microbial populations found within oil-contaminated ecosystems (96), including the deep subsurface plume that formed during the Deepwater Horizon spill (97).

Our findings here also reveal the potential involvement of the sulfur cycle in facilitating hydrocarbon degradation and potentially other ecosystem functions. The Alphaproteobacteria contain *soxXYZABC* genes, which are involved in sulfur oxidation, suggesting that there could be sulfur inputs to the water column in the FSC, such as may be derived from the release of produced waters from oil extraction in this region and adjacent waters of the North Sea. Indeed, sulfur compounds are abundant in many crude oils (98), but they are also inherent to the chemical structure of some synthetic chemical dispersants (e.g., Corexit) that are used to combat large oil spills at sea (99). Further, understanding these geochemical dependencies could inform fertilization strategies to enhance the biodegradation of specific compounds (96).

### Conclusions

In this study, we provide an experimental, genomic, and metabolic examination of the hydrocarbon-degrading baseline microbial communities from a subarctic region of the North Atlantic. Our findings highlight the dominance of bacteria capable of oil degradation, including novel genotypes with unique alkane degradation strategies. These bacteria, such as uncultured Alphaproteobacteria related to *Hyphomonas*, *Celeribacter*, *Lentibacter*, *Teteyamarina*, and *Pseudophaeobacter*, which are known hydrocarbon degraders, possess a previously undescribed mechanism for alkane hydroxylation in the absence of rubredoxins, which are essential for electron transfer. The prevalence of *Lentibacter* in the water column and hydrocarbon enrichments suggests they play an active role in purging the FSC waters of hydrocarbons. Finally, we demonstrated that *Dokdonia* assimilates and has pathways for *n*-hexadecane utilization in the absence of rubredoxins. This study highlight a network pathways spread across bacterial communities codes the metabolisms for hydrocarbon cycling. Collectively, these bacteria can coordinate the complete biodegradation of aliphatic and aromatic hydrocarbons in the event of an acute major oil spill or slow and continual chronic spillage. This study provides important insights into the microbial diversity and genetic mechanisms of hydrocarbon-degrading bacteria in a region with an active oil industry presence but which shows no signs of oil spillage. These findings have implications for understanding the potential for bioremediation strategies in areas affected by oil spills and other hydrocarbon contaminants. Overall, our findings have significant implications for managing and mitigating oil spills in subarctic deepwater regions. By understanding the microbial communities and their responses to environmental changes, we can develop more effective strategies to minimize the impact of oil spills on these sensitive ecosystems.

## Data Availability

The following accession numbers were submitted to GenBank for ^13^C-enriched DNA in SIP experiments with n-hexadecane (KY515280, KY515282, KY515284, KY515286–KY515288, KY515291–KY515294). The MAGs are submitted under project ID PRJNA816150. Raw data and annotations are provided in IMG/MER under project IDs 3300039448, 3300039456, 3300039449, and 3300040958.

## SUPPLEMENTAL MATERIAL

Supplemental Figures msystems.00619-23-s0001.docx Figures S1 to S13.

Table S1 msystems.00619-23-s0002.docx Quantitative PCR primers.

Table S2 msystems.00619-23-s0003.xlsx Overview of the metagenome assembly.

Table S3 msystems.00619-23-s0004.xlsx Sequencing data and reports from JGI.

Table S4 msystems.00619-23-s0005.xlsx MAG statistics.

Table S5 msystems.00619-23-s0006.xlsx Relative abundance of MAGs in the metagenomes.

Table S6 msystems.00619-23-s0007.xlsx GTDB-tk taxonomy.

Table S7 msystems.00619-23-s0008.xlsx KEGG-based functional annotation of the 42 MAGs from the n-hexadecane SIP enrichments.

Table S8 msystems.00619-23-s0009.xlsx KEGG-based functional annotation of the enzymes that are only present in our MAGs that belong to the *Oceanobacter*-related bacteria and are absent in the *Oceanobacter* reference genomes.

Tables S9 and S10 msystems.00619-23-s0010.xlsx Best hits found in CANT-HYD database and to the reference genome of *Alcanivorax dieselolei*.

ASM does not own the copyrights to Supplemental Material that may be linked to, or accessed through, an article. The authors have granted ASM a non-exclusive, world-wide license to publish the Supplemental Material files. Please contact the corresponding author directly for reuse.
