# Supplementary material for: Exploring novel alkane-degradation pathways in uncultured bacteria from the North Atlantic Ocean
Source: mSystems. 2023 Sep 13;8(5):e00619-23. doi: 10.1128/msystems.00619-23 (PMC10654063; doi:10.1128/msystems.00619-23)
Supplement: Supplemental Figures — Figures S1 to S13. [file msystems.00619-23-s0001.docx]

**Supplementary Figures**

**Exploring novel alkane-degradation pathways in uncultured bacteria from the North Atlantic Ocean**

Mirna Vázquez Rosas Landa^1,4^, Valerie De Anda^1,3^, Robin R. Rohwer^3^, Angelina Angelova^2^, Georgia Waldram^2^, Tony Gutierrez^2*^ and Brett J. Baker^1,3*^

1 Department of Marine Science, University of Texas Austin, Marine Science Institute, Port Aransas, Texas 78373, USA

2 Institute of Mechanical, Process and Energy Engineering (IMPEE), School of Engineering and Physical Sciences, Heriot-Watt University, Edinburgh EH14 4AS, UK.

3 Department of Integrative Biology, University of Texas at Austin

4 Unidad Académica de Ecologia y Biodiversidad Acuática, Instituto de Ciencias del Mar y Limnologia, Universidad Nacional Autónoma de Mexico, 04510 Mexico City, Mexico.

*Correspondence: [acidophile@gmail.com](mailto:acidophile@gmail.com), [Tony.Gutierrez@hw.ac.uk](mailto:Tony.Gutierrez@hw.ac.uk)


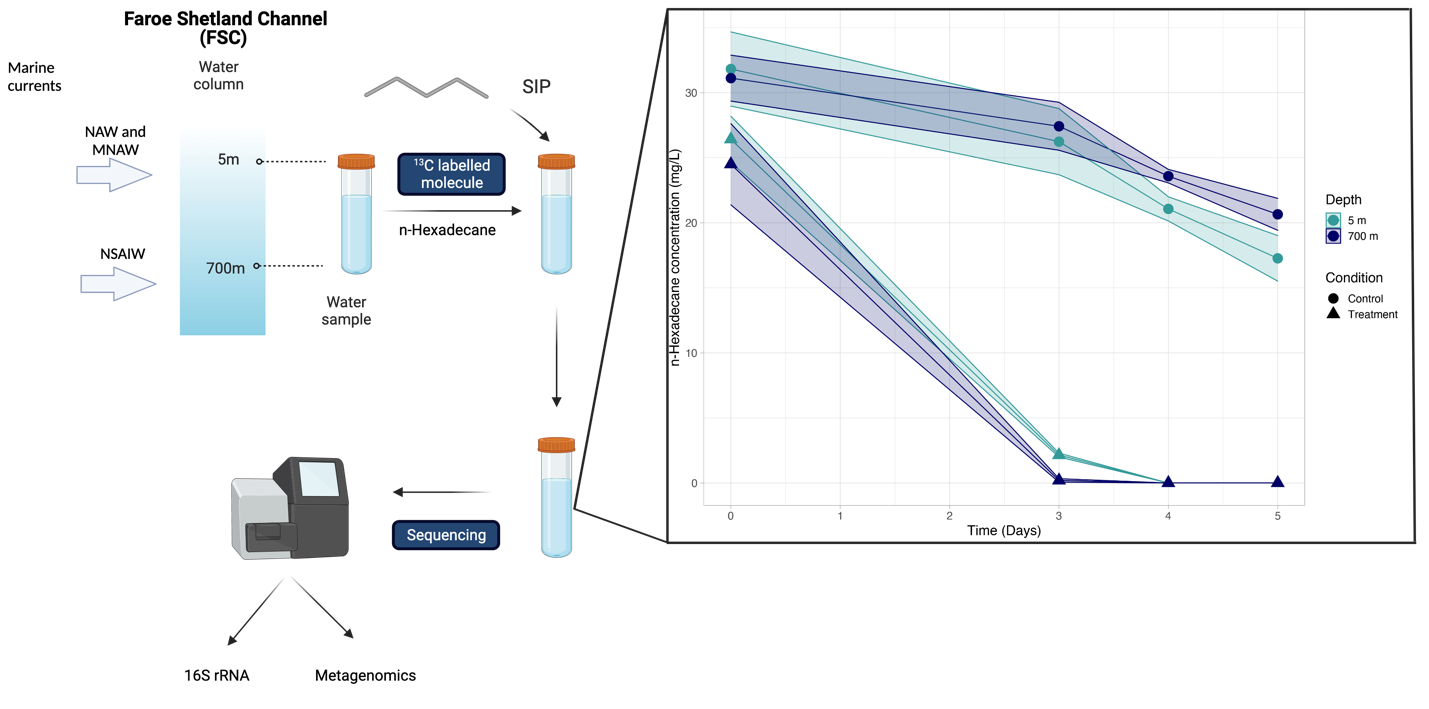


**Supplementary Figure S1.** **Overview of the SIP experiment labeled with ^13^C *n*-hexadecane.** We took samples at two different depths (5m and 700m) for the SIP enrichment experiments. The graph shows the biodegradation of *n*-hexadecane in incubations of the 5m (light blue) and 700m (dark blue) depth seawater as measured by GC-MS during the experiment. The endpoint for these SIP incubations was determined to be five days. Each data point is the mean results from triplicate flasks ± standard deviations. Triangles represent live cultures (non-acid treated); circles represent acid-inhibited controls. Shades represent the standard deviation. Finally, we extracted DNA to perform metagenomics and 16S rRNA clone library amplification, after complete degradation (see methods).


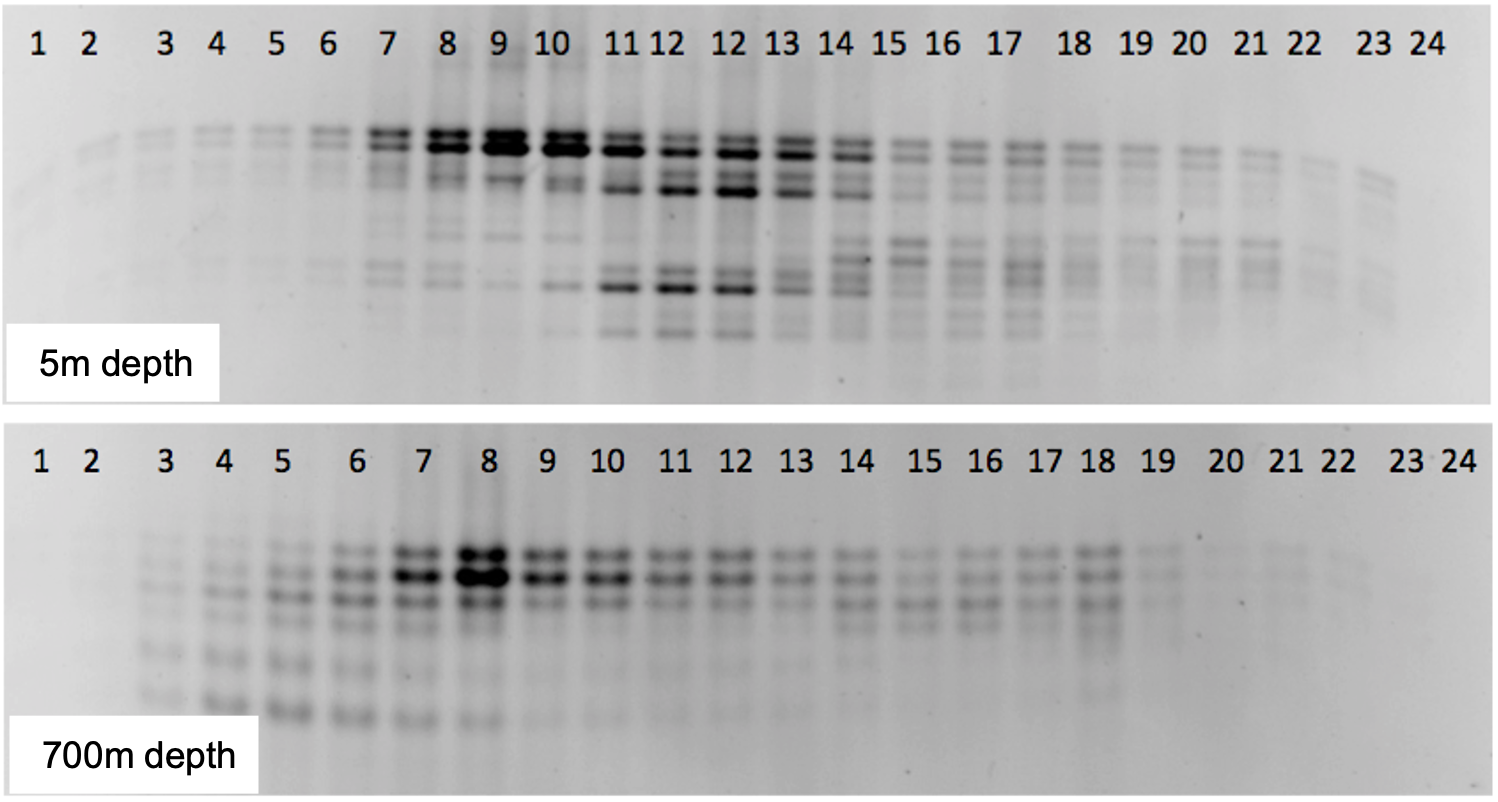


**Supplementary Figure S2.** Distribution of the ‘heavy’ and ‘light’ DNA in separated SIP fractions from the incubations using surface (5 m depth; top panel) and subsurface (700 m depth, bottom panel) water, as analyzed by DGGE of bacterial PCR products with decreasing densities from left to right.


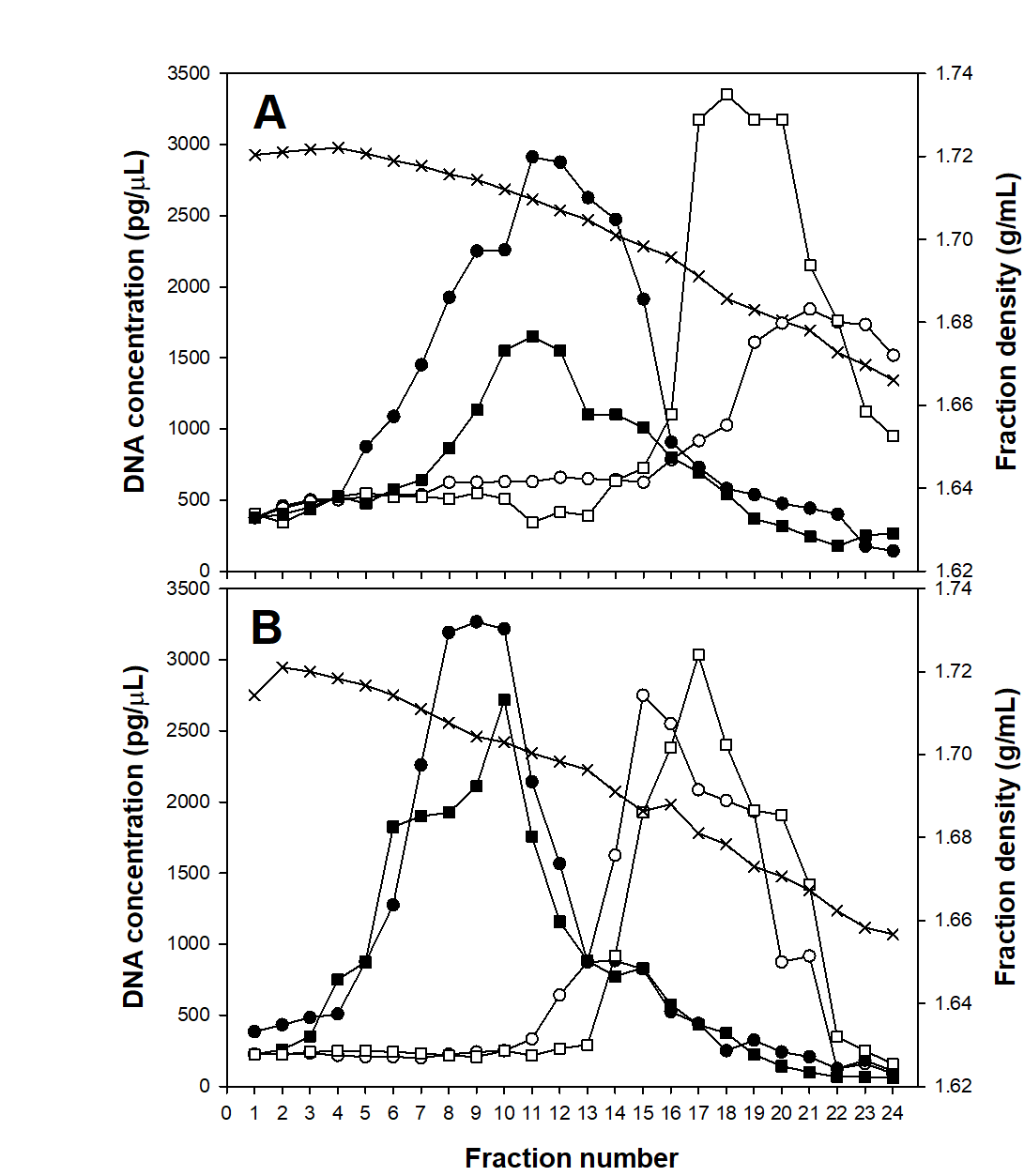


**Supplementary Figure S3.** DNA concentration quantified in fractions from labeled [^13^C]-hexadecane and unlabelled [^12^C]-hexadecane incubations using the sea surface (5 m depth) (A) and subsurface (700 m depth) (B) water. The duplicate ^13^C SIP incubations are represented by the curves with solid symbols, whereas the respective unlabelled incubations are represented by the curves with open symbols. The density of each fraction (represented with cross symbols) is also shown to demonstrate the successful formation of a density gradient during isopycnic ultracentrifugation. For the ^13^C-incubation using the surface water, fractions 7-10 were combined (see also Suppl. Fig. S2A), and for the ^13^C-incubation with the subsurface water, fractions 7-9 were combined (see also Suppl. Fig. S2B).


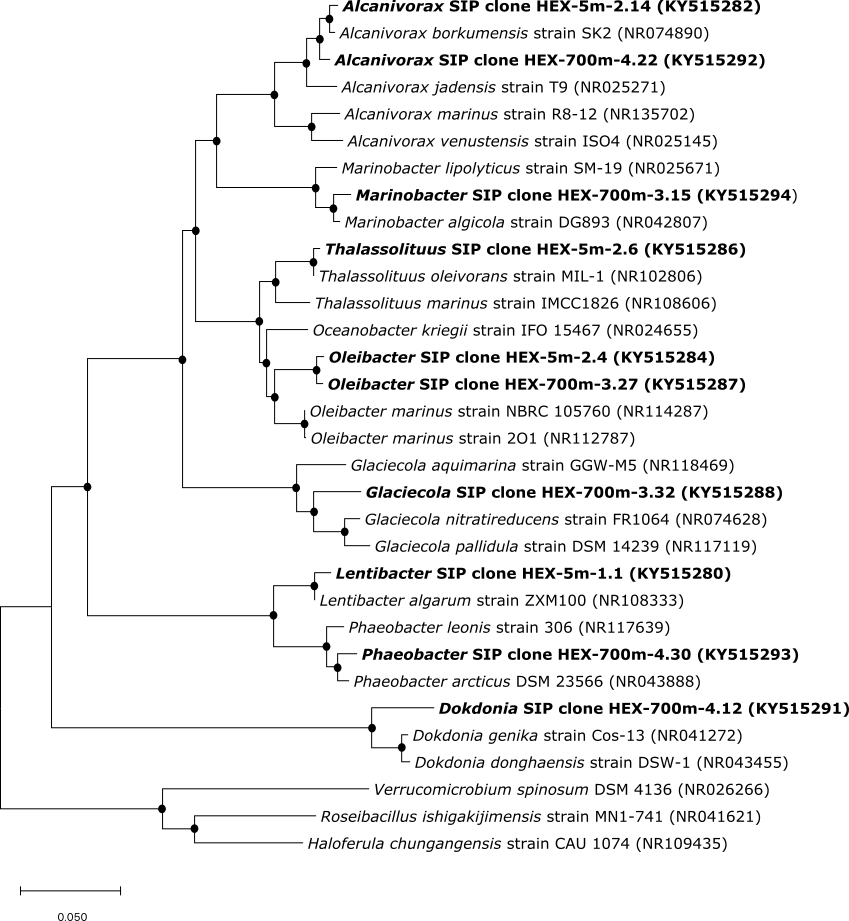


**Supplementary Figure S4.** 16S rRNA phylogenetic tree of the ^13^C-enriched community.


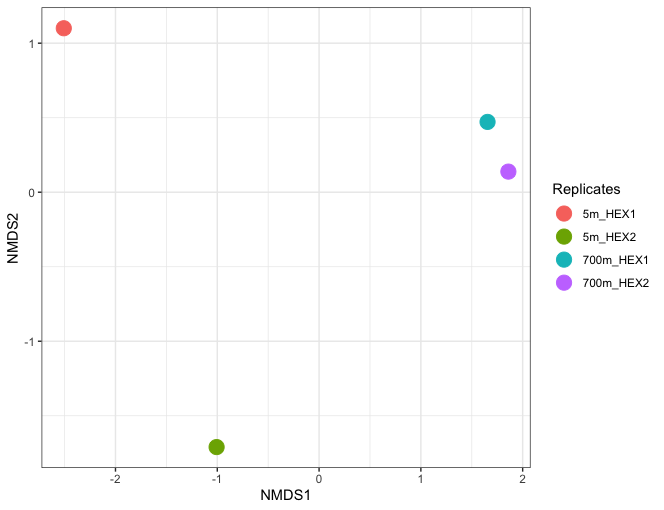


**Supplementary Figure S5.** The non-metric multidimensional scaling (NMDS) analysis of the community structure among replicates. The plot demonstrates that the community composition differs significantly among the replicates collected at 5m depth. The analysis was performed using the 'vegan' package in R software, and the input data was based on the abundance of different bacterial genera in the samples. The Euclidean distance was used as a measure of dissimilarity, and the analysis was performed on complete cases. The plot indicates that the replicates at 5m depth form distinct clusters, while the replicates collected at 700m depth do not exhibit such distinct clustering. This suggests that the community composition at 5m depth is more variable among replicates than at 700m depth.


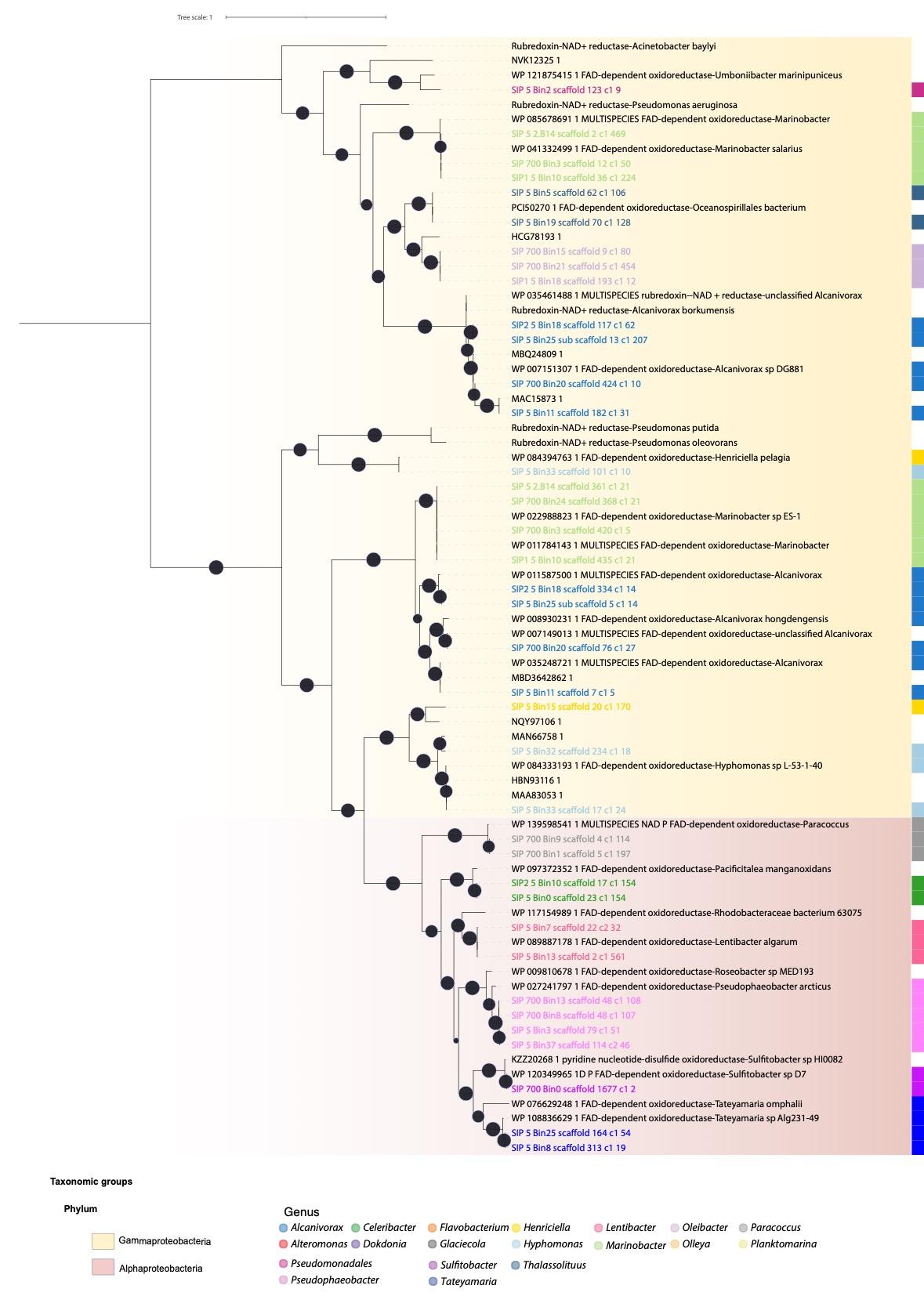


**Supplementary Figure S6.** Maximum-likelihood-based phylogenetic tree of AlkT. Black circles within the branches indicate bootstrap values >80%, where the smallest circle equals 80% and the biggest 100%. Clades are color-coded according to the taxonomic group to which each sequence belongs: yellow, Gammaproteobacteria; pink, Alphaproteobacteria; purple, Bacteroidetes. Label colors represent the genera to which each sequence belongs.


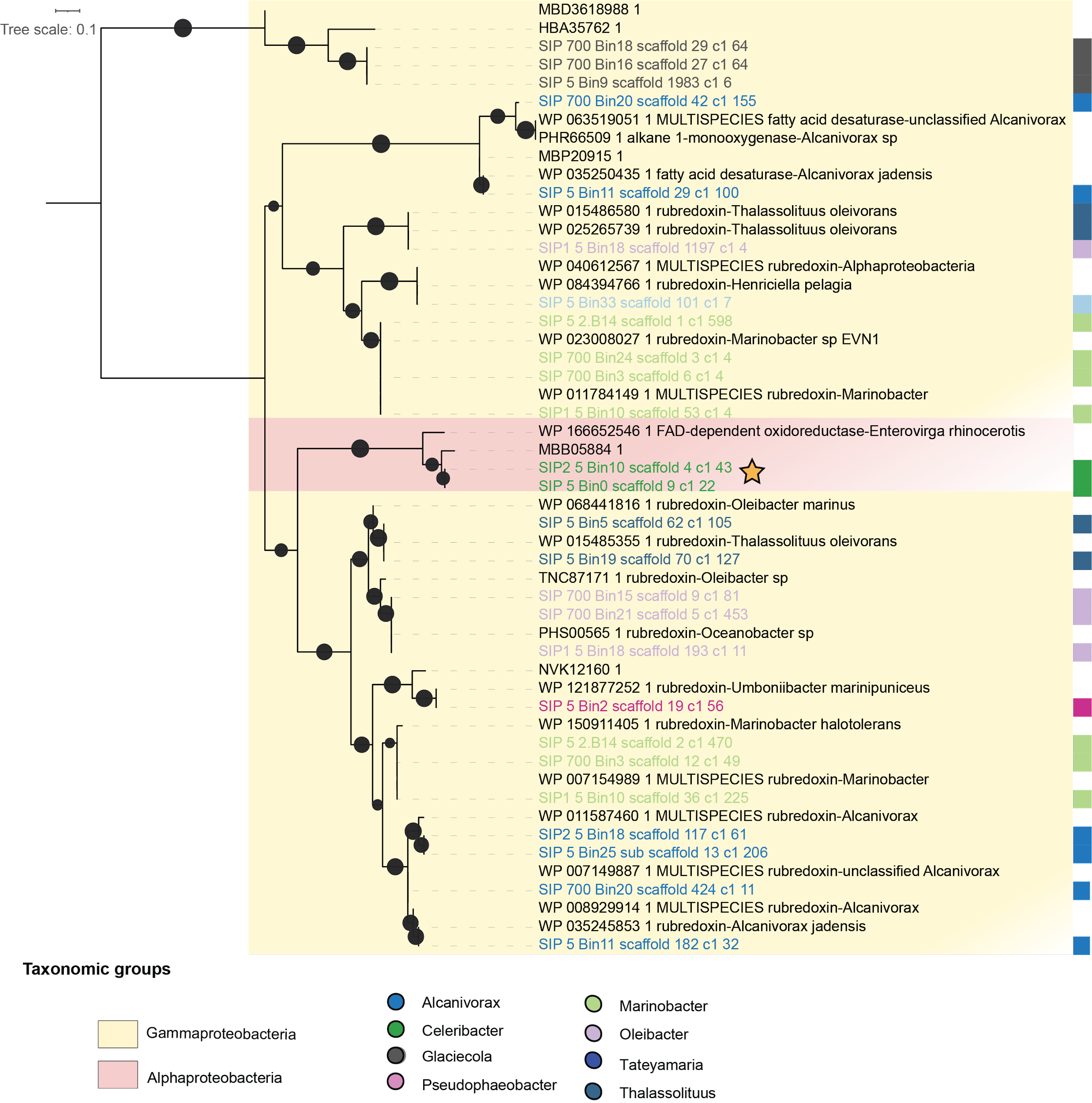


**Supplementary Figure S7.** Maximum-likelihood-based phylogenetic tree of AlkG. Black circles within the branches indicate bootstrap values >80%, where the smallest circle equals 80% and the biggest 100%. Clades are color-coded according to the taxonomic group to which each sequence belongs: yellow, Gammaproteobacteria; pink, Alphaproteobacteria; purple, Bacteroidetes. Label colors represent the genera to which each sequence belongs. The stars show the sequences with fusion domains.


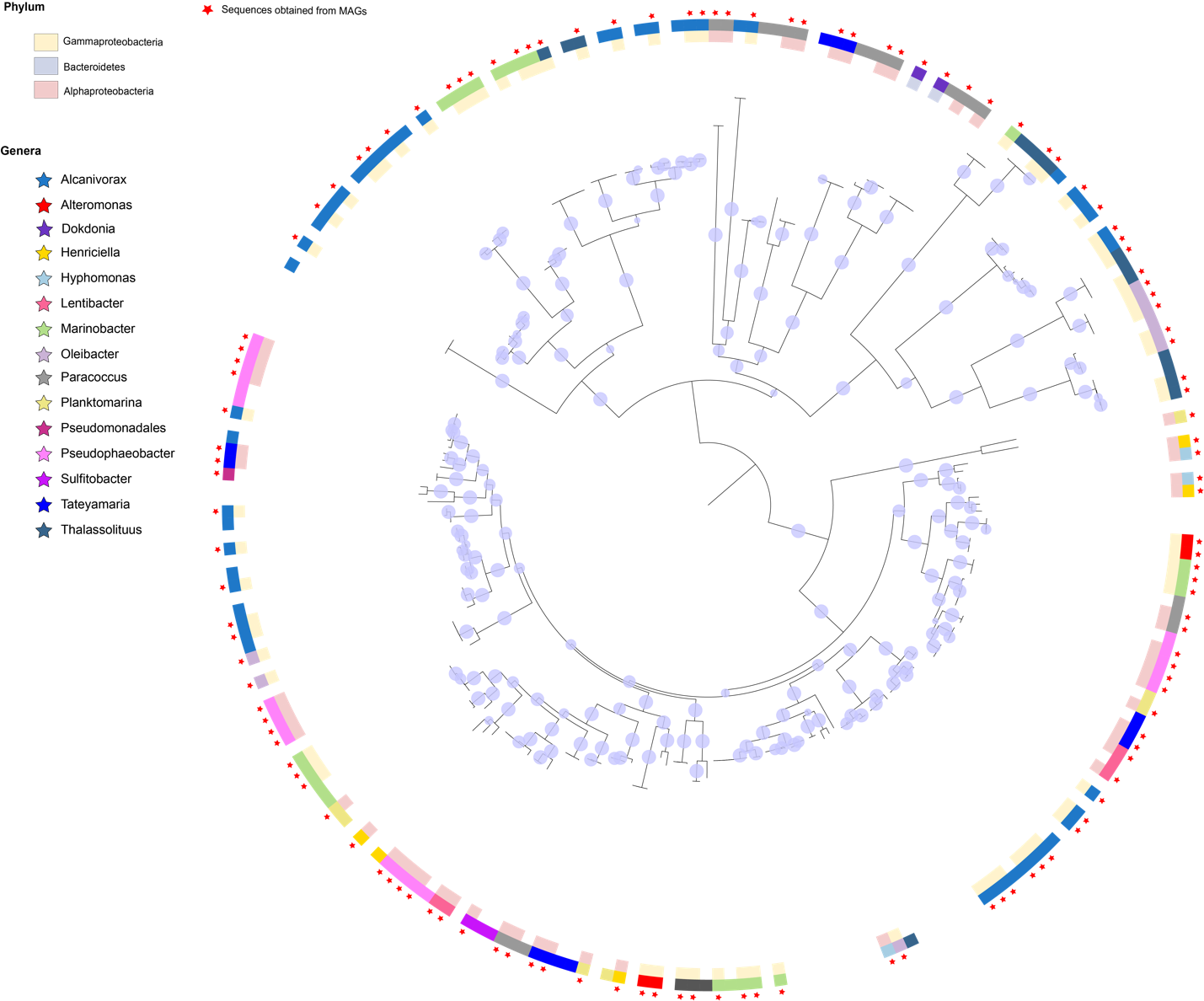


**Supplementary Figure S8.** Maximum-likelihood-based phylogenetic tree of AlkJ. Bootstrap values greater than 80% are represented by grey circles, with the smallest circle indicating 80% and the largest indicating 100%. Taxonomy is indicated by label colors, and sequences obtained from metagenome-assembled genomes (MAGs) are denoted by stars. We observe in the inner circle that each phylum in the tree includes at least one MAG containing the necessary protein domain for the enzyme function. Branches with no color represent reference sequence.


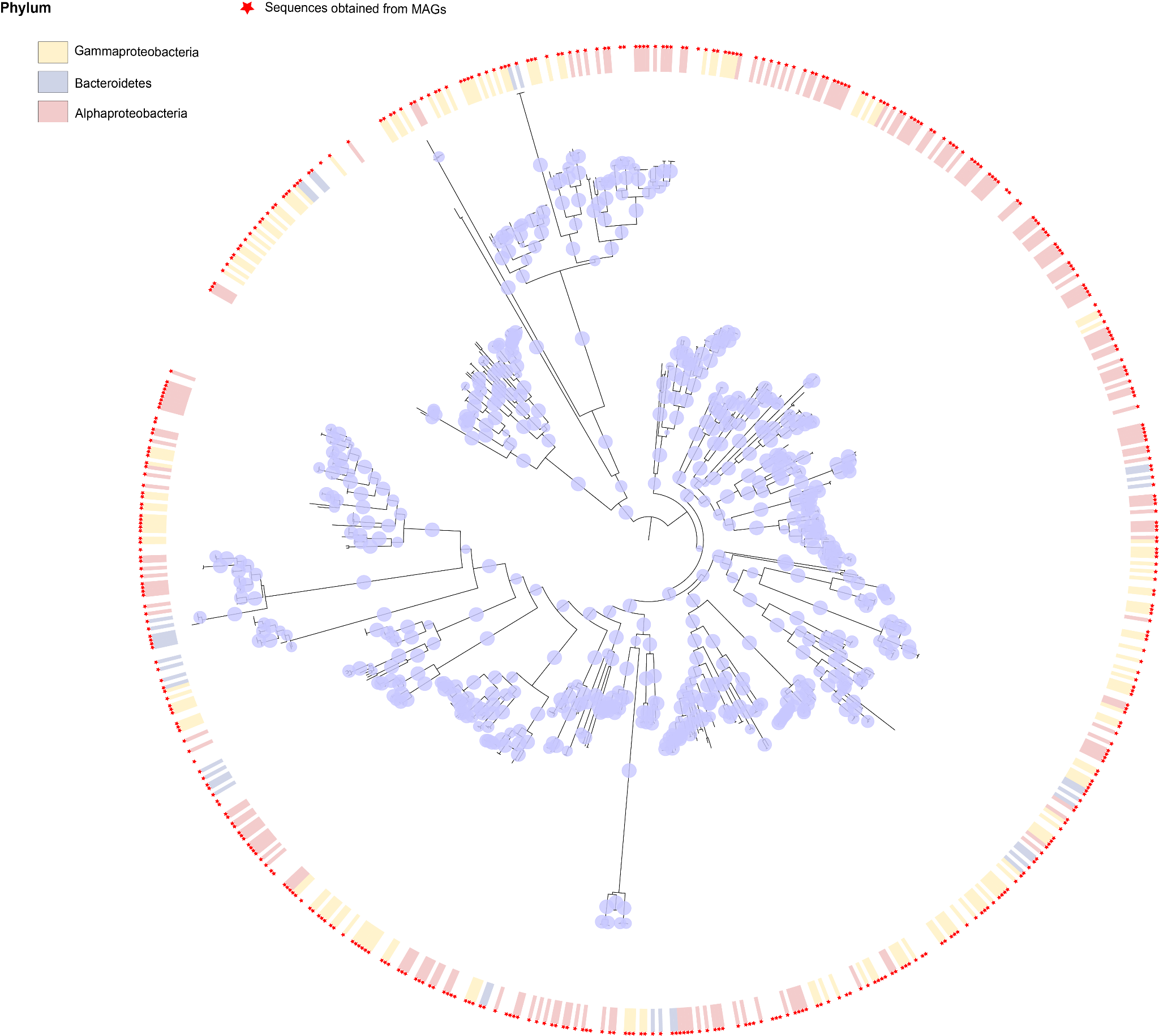


**Supplementary Figure S9.** Maximum-likelihood-based phylogenetic tree of AlkH. Bootstrap values greater than 80% are represented by grey circles, with the smallest circle indicating 80% and the largest indicating 100%. Taxonomy is indicated by label colors, and sequences obtained from metagenome-assembled genomes (MAGs) are denoted by stars. We observe in the inner circle that each phylum in the tree includes at least one MAG containing the necessary protein domain for the enzyme function. Branches with no color represent reference sequences.

Recently the *Oleibacter* genus has been proposed to be renamed *Thalassoulitus* based on its gene content. Our phylogenetic analysis grouped *Oleibacter* and *Thalassolituus* into two different clusters (Main text Fig. 2). However, this distinction may be made by the need for other reference genomes of *Thalassolituus*. Furthermore, our MAGs also cluster with the genome of an *Oceanobacter*. Therefore, we analyzed their gene content to ensure our *Oleibacter* MAGs were not *Oceanobacter* and to see its relation with other *Thalassolituus* and *Oleibacter* genomes. Our analysis showed that the reference genome labeled as *Oceanobacter* shares more genes with the *Oleibacter* MAGs than with the other *Oceanobacter* genomes (Suppl. Fig 10). Therefore, the genome should be renamed to *Oleibcater* (*Thalassolituus*). Finally, the gene content analysis showed that our MAGs also cluster among other *Thalassolituus*, supporting the fact that *Oleibacter* should be renamed to *Thalassolituus*.

**
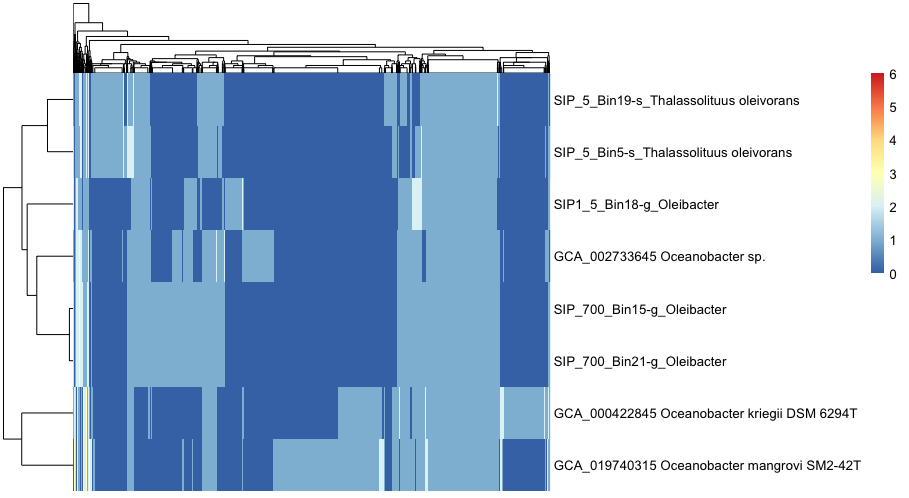
**

**Supplementary Figure S10.** Heatmap showing the 7,230 proteins shared by our MAGs and the Oceanonabcter reference genomes.

**
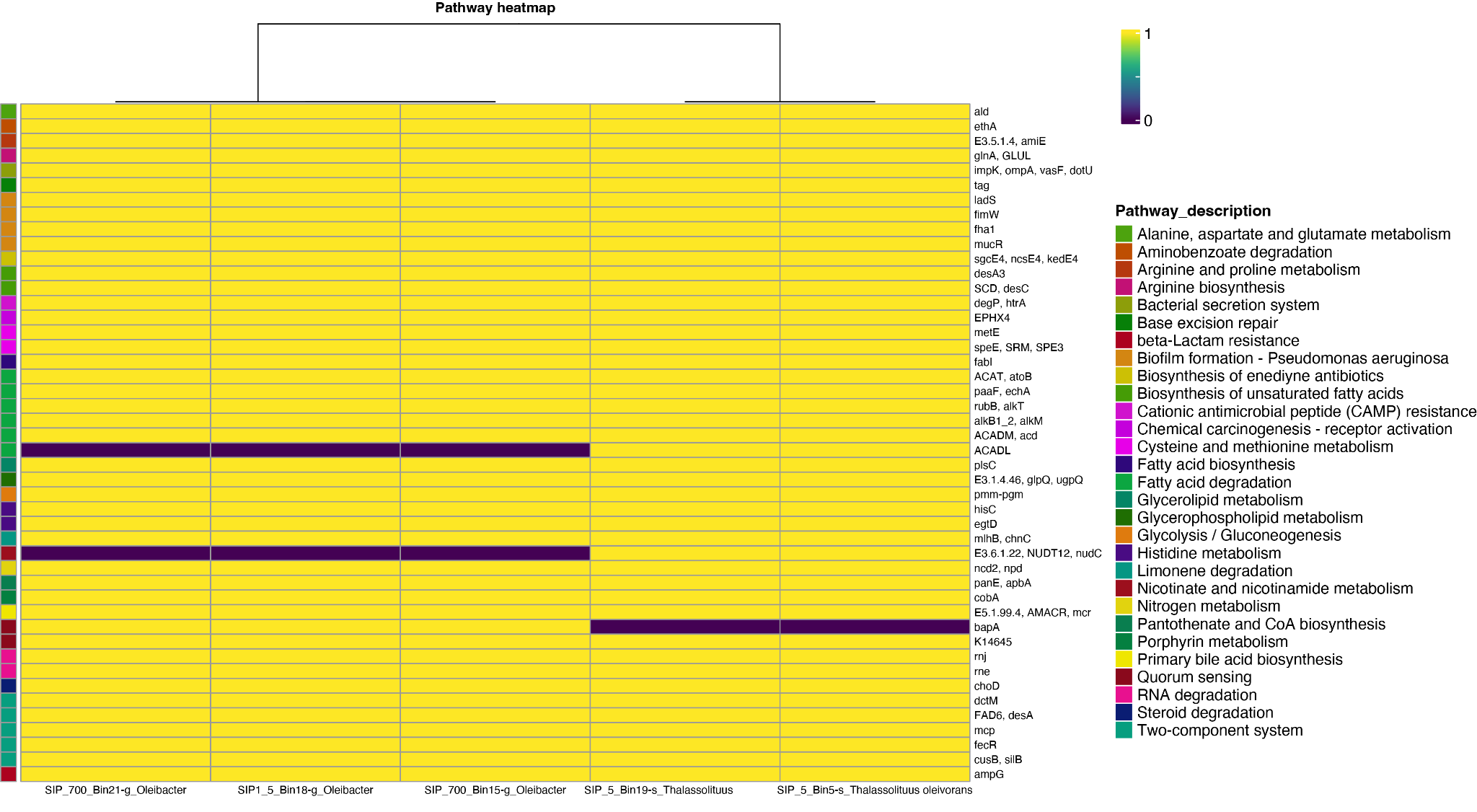
**

**Supplementary Figure S11:** Heatmap of unique gene functions and pathways present in our MAGs and absent in Oceanobacter. We identified 272 proteins not found in Oceanobacter but present in our MAGs. The heatmap displays the gene functions and pathways associated with these unique proteins.


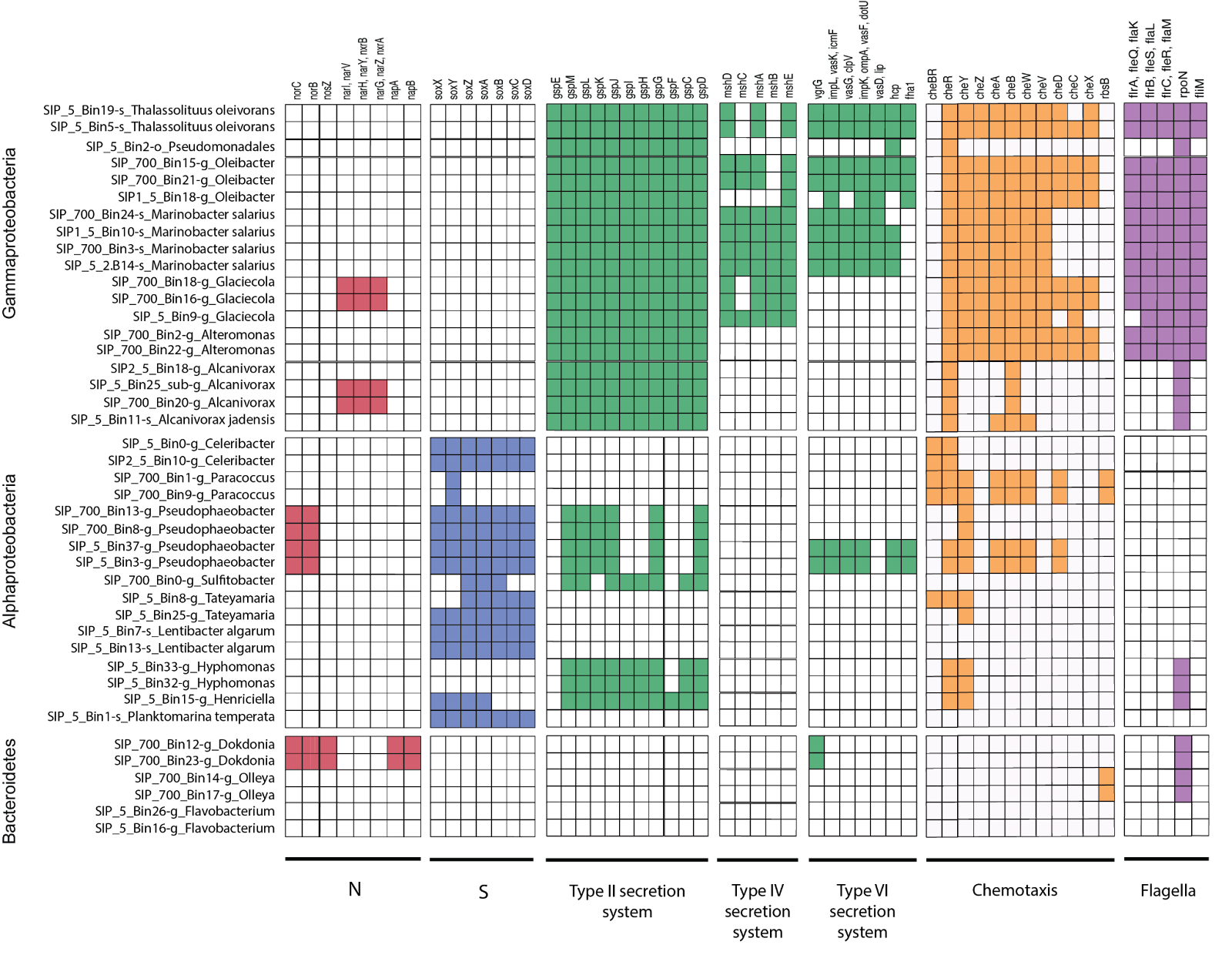


**Supplementary Figure S12.**  Distribution of genes related to other energy metabolism and community interactive functions in bacterial genomes from the FSC. The plot is horizontally divided into Gammaproteobacteria, Alphaproteobacteria and Bacteroidetes.


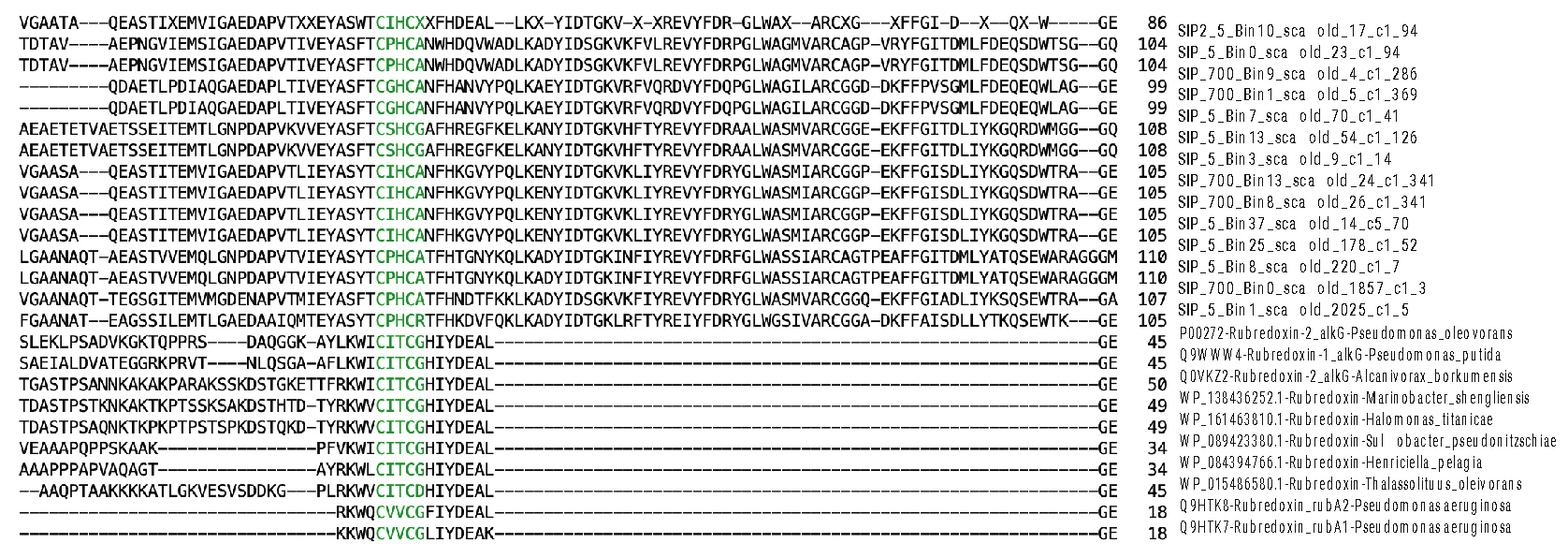


**Supplementary Figure S13.** The AlkG-like and AlkG protein alignment, letters highlighted in green show the shared motif.
