# Supplementary material for: Exploring novel alkane-degradation pathways in uncultured bacteria from the North Atlantic Ocean
Source: mSystems. 2023 Sep 13;8(5):e00619-23. doi: 10.1128/msystems.00619-23 (PMC10654063; doi:10.1128/msystems.00619-23)
Supplement: Table S1 — Quantitative PCR primers. [file msystems.00619-23-s0002.docx]

**Supplementary Table S1.** Quantitative PCR primers developed and used in this study.



Target Primer qPCR Amplicon

OTU name Primer sequence (5’ 3’) standard^a^ length




2.14 Alc-414f TTTCAGTAGGGAGGAAGGCT HEX-5m-2.14 78

Alc-490r AAATTAGCCGGTGCTTCTTCT

2.6 Thal-417f AGGGAGGAAAGGTTGTGTGTT HEX-5m-2.6 163

Thal-578r ACATCTCGCTTAACAAACAACC

1.1 Len-933f CAGGCTTTCCTTCGGGACATAG HEX-5m-1.1 108

Len-1039r AACTAGGGATGTGGGTTGCG

2.4 Ole-172f GGGCTTCGGCTCTCGCTAT HEX-5m-2.4 72

Ole-242r CCTTGGTAGGCCTTTACCCC

3.27 Ole-614f AACTGGCAAGCTAGAGTACAGT HEX-700m-3.27 114

Ole-726r TCAGTATCAGTCCAGGCAGTC

4.22 Alc-122f GGGGGATAACCTGGGGAAAC HEX-700m-4.22 87

Alc-207r ATCCATCAGCACAAGGTCCG

3.15 Mar-702f GCGGCACTCTGGACCAATAC HEX-700m-3.15 118

Mar-818r CTTCAAGAGTCCCAACGGCT

4.3 Phae-1105f TGTCCAAGATTCCCCACTGC HEX-700m-4.3 160

Phae-1264r TTTATCGGAGAAGGGTCGGC

3.32 Glac-713f ACCCACACTTTCGCACATGA HEX-700m-3.32 121

Glac-883r GTCTTGGAGAGGGGAGTGGA

4.12 Dok-730f GACGAAAGCGTAGGTAGCGA HEX-700m-4.12 76

Dok-805r CCACACAGCTAGTAACCATCG



^a^ Representative clone sequences from which plasmid DNA was used to generate standard curves. Each plasmid was linearized with EcoRV.
